# Adiponectin pathway activation dampens inflammation and enhances alveolar macrophage fungal killing via LC3-associated phagocytosis

**DOI:** 10.1371/journal.ppat.1012363

**Published:** 2025-03-17

**Authors:** Sri Harshini Goli, Joo-Yeon Lim, Nese Basaran-Akgul, Steven P. Templeton

**Affiliations:** 1 Department of Microbiology and Immunology, Indiana University School of Medicine-Terre Haute, Terre Haute, Indiana, United States of America; 2 Department of Biology, Indiana State University, Terre Haute, Indiana, United States of America; Rutgers New Jersey Medical School, UNITED STATES OF AMERICA

## Abstract

Although innate immunity is critical for antifungal host defense against the human opportunistic fungal pathogen *Aspergillus fumigatus*, potentially damaging inflammation must be controlled. Adiponectin (APN) is an adipokine produced mainly in adipose tissue that exerts anti-inflammatory effects in adipose-distal tissues such as the lung. We observed increased mortality and increased fungal burden and inflammation in neutropenic mice with invasive aspergillosis (IA) that lack APN or the APN receptors AdipoR1 or AdipoR2. Alveolar macrophages (AMs), early immune sentinels that detect and respond to lung infection, express both receptors, and APN-deficient AMs exhibited an inflammatory phenotype that was associated with decreased fungal killing. Pharmacological stimulation of AMs with AdipoR agonist AdipoRon rescued deficient killing in APN-/- AMs and was dependent on the presence of either receptor. Finally, APN-enhanced fungal killing was associated with increased activation of the non-canonical LC3 pathway of autophagy. Thus, our study identifies a novel role for APN in LC3-mediated killing of *A.fumigatus*.

## Author summary

*Aspergillus fumigatus* is a human fungal pathogen that causes an often-fatal invasive infection of the lung in immune compromised individuals, a population that is increasing. Since current antifungal drugs have limited efficacy, it is important to identify pathways that may be pharmaceutically targeted to complement existing therapies. Adiponectin (APN) is an anti-inflammatory intercellular cytokine messenger produced mainly in adipose tissue that protects against invasive aspergillosis. Alveolar macrophages are early immune sentinel cells in the lung, and we report that AMs from mice lacking APN exhibit an inflammatory phenotype and reduced killing of *A. fumigatus* spores that is improved when AMs are treated with a drug (AdipoRon) that simulates APN binding to its receptors AdipoR1 and AdipoR2. Furthermore, APN was associated with activation of LC3-associated phagocytosis, a mechanism of fungal killing important for host defense against *A. fumigatus* infection. Thus, we identify therapeutic potential of the APN pathway in stimulation of immune-mediated fungal killing and treatment of fungal infection.

## Introduction

The human opportunistic fungal pathogen *Aspergillus fumigatus* is the primary etiologic agent of invasive pulmonary aspergillosis (IA), a frequently severe infection with a high mortality rate [1]. Effective treatment options for IA remain limited, despite increases in the at-risk population, and fungal resistance to existing antifungal drugs is increasing. Furthermore, although an underlying immune deficiency renders individuals susceptible to IA, poor disease outcomes are associated with detrimental inflammatory pathology [2,3]. It is thus critical to enhance antifungal immunity while limiting immune pathology to complement existing antifungal therapies in IA patients.

Adiponectin (APN) is an anti-inflammatory adipokine produced mainly in adipose tissue and expressed at high levels in lean and healthy individuals, whereas in obese individuals, adipose tissue is inflamed, and APN production and secretion are inhibited [4–6]. Numerous studies have demonstrated a protective effect for APN in autoimmune and inflammatory diseases, and immune-mediated clearance of *Listeria monocytogenes* was hampered in obese and APN-deficient mice due to detrimental inflammation and hematopoietic dysfunction [7]. More recently, we demonstrated increased mortality, fungal burden, lung inflammatory cytokine production and eosinophil recruitment in APN-deficient mice with IA [8] However, eosinophils did not play a significant role in the pathology of APN-deficiency, as neutropenic mice deficient in both APN and eosinophils exhibited only delayed, but not decreased mortality, suggesting other unidentified mechanisms of APN-mediated protection in IA.

Alveolar macrophages (AMs) are the primary immune sentinel of lung airways, and their early responses to inhaled fungal conidia are critical for disease outcomes [9–11], particularly in immunocompromised hosts [12,13]. In our previous study, we observed increased production of the pro-inflammatory cytokine TNF in APN-deficient AMs [8], suggesting that these cells contribute to the inflammatory phenotype of APN-/- mice with IA. In this study, we aimed to determine how the adiponectin pathway regulates responses of AMs to *A. fumigatus* infection. We report increased production of inflammatory cytokines, an elevated inflammatory phenotype, and reduced uptake and killing of conidia in APN-deficient AMs that is increased by stimulation with the APN receptor agonist AdipoRon. Furthermore, APN stimulation was associated with increased AM activation of the non-canonical autophagy LC3 pathway in conidia-containing phagosomes, thus identifying a novel role for APN in promoting LC3-associated phagocytosis (LAP) in antifungal immunity.

## Results

### Increased mortality, fungal burden, and inflammatory pathology in APN pathway-deficient mice with IA.

**Fig 1:**
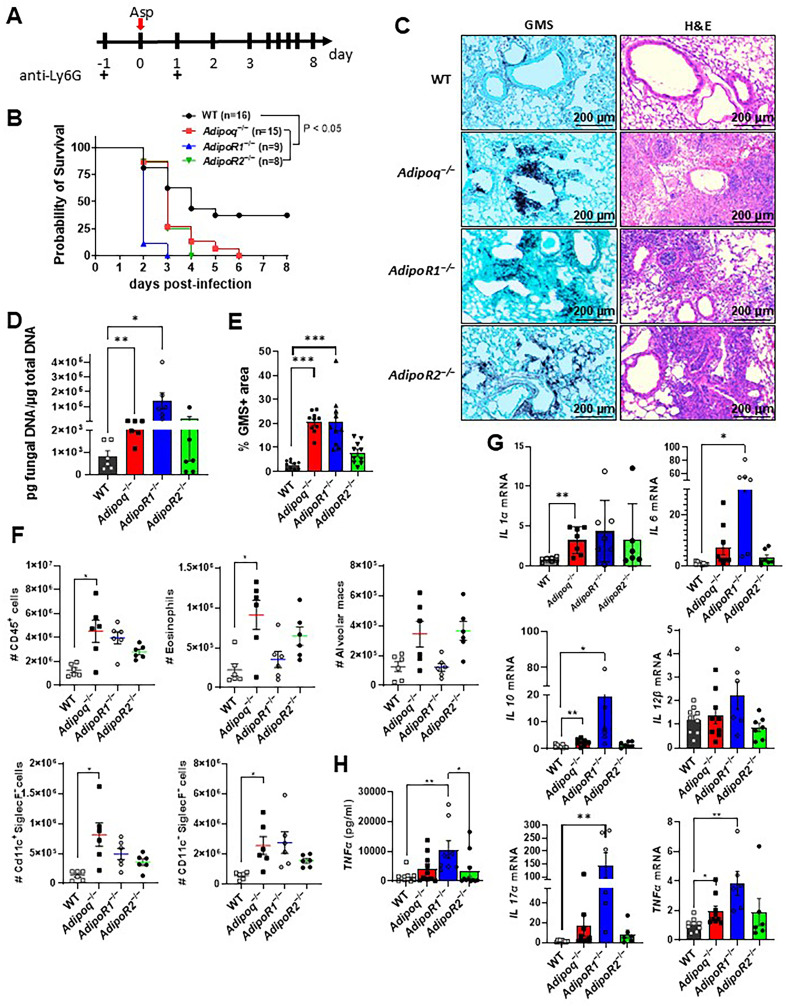
Increased mortality, fungal burden, and inflammation in APN pathway-deficient mice with invasive aspergillosis. Wild-type (C57BL/6), *Adipoq*^−/−^, *AdipoR1*^−/−^, and *AdipoR2*^−/−^ mice were neutrophil-depleted and involuntarily aspirated a suspension of *A. fumigatus* conidia and followed for survival or sacrificed on day 3 post-infection as described in *Materials and Methods*. A. Timeline for neutropenic mouse model of infection. B. Survival. C. Representative GMS and H&E lung sections. D. Fungal burden determined by quantitative PCR of fungal DNA from lung homogenates. E. Fungal burden determined by quantification of GMS staining. F. Total number of CD45^+^ cells, eosinophils (CD45^+^Ly6G^−^CD11c^−^SiglecF^+^), AMs (CD45^+^Ly6G^−^CD11c^+^SiglecF^+^), CD11c^+^SiglecF^−^ (CD45^+^Ly6G^−^ CD11c^+^SiglecF^−^), and CD11c^−^SiglecF^−^ (CD45^+^Ly6G^−^CD11c^−^SiglecF^−^) cells isolated from the mice with IA as determined by flow cytometry. G. qRT-PCR analysis for mRNA expression of the indicated cytokines. H. TNFα secretion in BALF quantified by ELISA. Data are a summary of two independent experiments. *p < 0.05, **p < 0.01, ***p < 0.001.

Previously, we reported that APN inhibits inflammatory lung pathology during IA, as neutropenic mice lacking APN exhibited increased mortality, fungal burden, and inflammation [8]. Since APN-deficient (*Adipoq-/-)* strains vary in their metabolic phenotypes [14–16], we confirmed the deficiencies in fungal clearance and inflammatory phenotype of APN-deficient mice from a second strain using our neutropenic model of IA (obtained from Dr. Philipp Scherer) (**S1 Fig**), and observed similar increases in fungal burden (**S1**B**-****S1D Fig**), inflammatory cytokines and inflammatory cell recruitment (**S1**E-F and **S1H-I Fig**) while only survival and *AdipoR1* transcription were not significantly different from wild-type (WT) C57BL/6 controls (**S1**A and **S1G Fig**, respectively). We also aimed to determine the contribution of the canonical adiponectin receptors AdipoR1 and AdipoR2 to IA pathology, and thus infected neutrophil-depleted WT, Adipoq-/-, *AdipoR1-/-* and *AdipoR2-/-* mice to compare mortality, pathology, fungal burden, inflammatory cell recruitment and cytokine production at day 3 post-infection (**Fig 1A**). In contrast to WT mice (with 50% mortality), mortality was 100% in APN-, AdipoR1-, and AdipoR2-deficient mice, with mortality beginning on day 2 post-infection and reaching 100% by day 6 p.i. (**Fig 1B**), with extensive lung hyphal growth and peri-bronchoalveolar inflammation (**Fig 1C**) and increased fungal burden as measured by qPCR of fungal DNA (**Fig 1D**) and image quantification of fungal GMS staining (**Fig 1E**), most significantly in APN- and AdipoR1-deficient mice. CD45+ Inflammatory cells were increased in the bronchoalveolar lavage fluid (BALF), most significantly (including eosinophils, CD11c^+^SiglecF^-^ cells and CD11c^-^SiglecF^-^ cells) in APN-deficient mice (**Fig 1F**), while lung inflammatory cytokines IL-1a, IL-6, TNF, IL-12b, and IL-17A and the anti-inflammatory cytokine IL-10 were increased, most significantly in AdipoR1-deficient mice (**Fig 1G** and **1H**). In summary, adiponectin-pathway deficiency is associated with increased mortality, lung fungal burden, and inflammatory pathology, cell recruitment, and cytokine production. Furthermore, disrupted signaling through either AdipoR1 or AdipoR2 resulted in increased IA severity.

### Alveolar macrophages (AMs) from adiponectin pathway-deficient mice exhibit an inflammatory phenotype.

**Fig 2:**
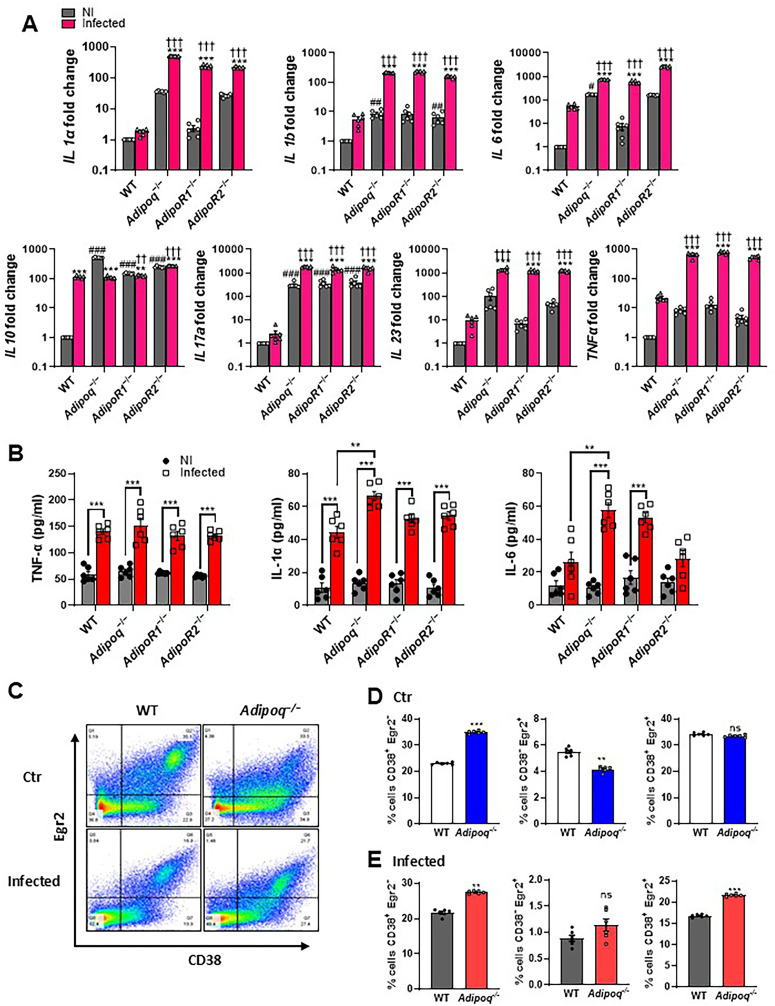
Alveolar macrophages from APN pathway-deficient mice exhibit an inflammatory phenotype. BALF cells from wild-type (C57BL/6), *Adipoq*^−/−^, *AdipoR1*^−/−^, and *AdipoR2*^−/−^ mice were collected in PBS/EDTA. For alveolar macrophage differentiation, total BALF cells were plated and cultured with GM-CSF. Alveolar macrophages were challenged with conidia in the ratio 1:9 (AMs: conidia) for 10 hours duration as per the details mentioned in *Materials and Methods*. A. qRT-PCR analysis for mRNA expression of the indicated cytokines. B. The indicated cytokines are quantified at the protein level by ELISA using ex vivo cultured alveolar macrophages. C. Flow cytometry staining of surface CD38 and intracellular Egr2. D-E. Quantification of (C), showing the proportion of M1, M2 and CD38^+^Egr2^+^ macrophages with CD38^+^Egr2^−^ (classical, inflammatory) or CD38^−^Egr2^+^ (alternative) phenotype in control group (D) and infection group (E). Data are a summary of two independently performed experiments. An asterisk (*) indicates a significant difference versus corresponding non-infected controls. A hash (#) indicates a significant difference between non-infected (NI) groups versus NI WT. A dagger (†) indicates a significant difference between infected groups versus infected WT. *p < 0.05, **p < 0.01, ***p < 0.001.

Previously, we observed increased intracellular TNF in AMs from APN-deficient mice with IA when compared to WT AMs [8], and we observed mortality as early as day 2 post-infection in APN-pathway-deficient mice with IA, suggesting a critical involvement of early immune effectors in protection from infection. As AMs contribute to early responses to inhalation of *A. fumigatus* [12], especially in immunocompromised hosts [13], we aimed to further examine the phenotype of AMs from APN pathway-deficient hosts using primary ex vivo cultures. We observed expression of both AdipoR1 and AdipoR2 in WT mice that decreased in lung tissue upon *A. fumigatus* infection, but increased in the BALF (**S2A Fig**), with the highest surface expression of AdipoR1 in AMs (**S2B Fig**). APN-pathway deficient AMs exhibited differential transcription of AdipoRs (**S2C Fig**), and infection of APN-/- AMs decreased AdipoR1 expression (**S2D Fig**). Furthermore, AMs maintained their surface expression of Siglec-F and CD11c through 25 days of culture (**S5A-B Fig**), and maintained transcription of the classical AM genes *Pparg* and *Car4* (**S5C Fig**).Transcription of IL-1α, IL-1β, IL-6, IL-17A, IL-23, and TNF was increased in APN pathway-deficient AMs when compared WT-derived AMs, while IL-10 was similar (**Fig 2A**), and secreted TNF, IL-1α, and IL-6 in the supernatant was increased in response to infection in all groups, with IL-1α and IL-6 increased in infected APN-deficient AMs compared to WT (**Fig 2B**). To investigate the inflammatory phenotype more specifically in ex vivo cultured APN-deficient AMs, we compared expression of the macrophage polarization markers CD38 (M1/classical) and Egr2 (M2/alternative) with wild type WT AMs [17]. In a resting state, a higher percentage of APN-deficient AMs were CD38+ and lower percentage were Egr2+ when compared to WT (**Fig 2C** and **2D**), while after 10 hours of infection with *A. fumigatus*, percentages of both CD38+Egr2- and CD38+Egr2+ cells increased, with no change in CD38-Egr2+ cells (**Fig 2C** and **2E**). These results suggest that APN pathway-deficient AMs exhibit an increased inflammatory phenotype compared to WT AMs.

### *A. fumigatus* uptake and killing is inhibited in APN-deficient AMs.

**Fig 3:**
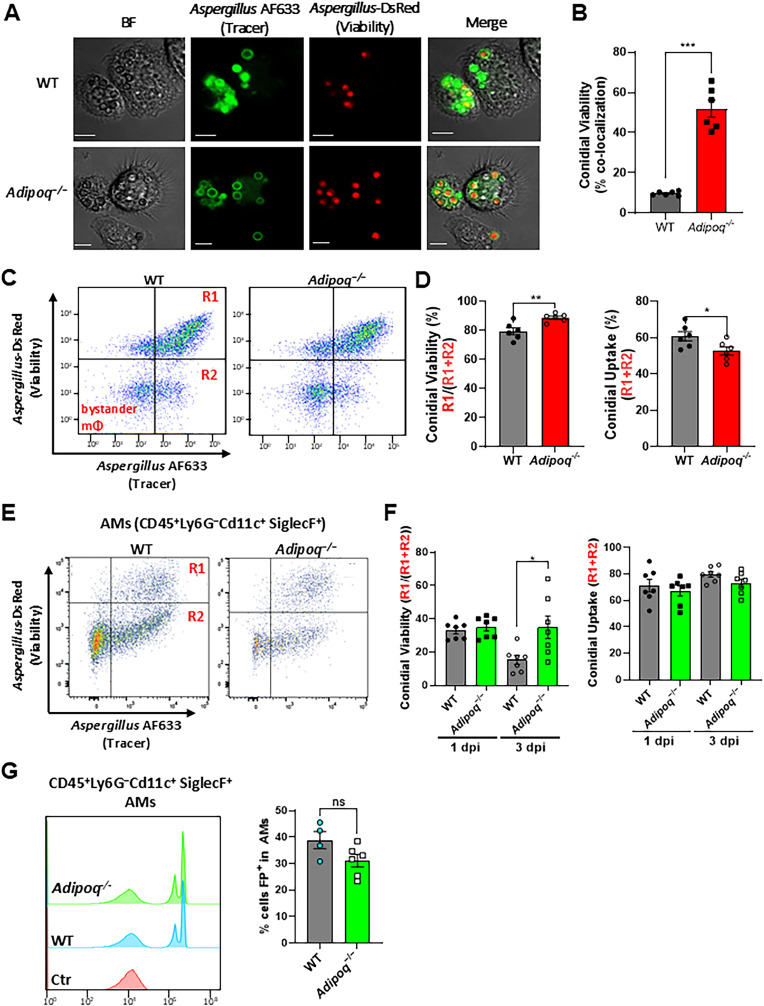
*A. fumigatus* conidial uptake and killing are inhibited in adiponectin-deficient AMs. AMs from wild-type or Adipoq-/- mice were infected ex vivo with FLARE conidia, or neutropenic mice were infected with FLARE conidia or exposed to latex beads in vivo and isolated as described in *Materials and Methods*. A. Microscopic analysis of uptake and killing of FLARE conidia with magnification 40x is depicted in Brightfield, Cy5, DsRed channels followed by the merged image. Scale bar is equal to 12 µm. B. Quantification of *Aspergillus* viability using microscopy images via calculation of co-localization in imageJ. C. Representative plots that display RFP and AF633 fluorescence intensity of *ex vivo* cultured alveolar macrophages where R1 represents live conidia inside the alveolar macrophages and R2 represents dead/killed conidia. D. Quantification of (C), showing *Aspergillus* viability (R1/(R1 + R2), left panel) and uptake (R1 + R2, right panel) in *ex vivo* cultured alveolar macrophages. E. Representative plots that display RFP and AF633 fluorescence intensity of AMs (CD45^+^Ly6G^−^CD11c^+^SiglecF^+^) *in vivo.* Mice of both groups were made immunocompromised and infected with 1-1.5 × 10^7^ conidia. BAL cells were harvested 1 day and 3 days later. F. Quantification of (E), showing *Aspergillus* viability (R1/(R1 + R2)) and uptake (R1 + R2) in alveolar macrophages in neutropenic mice at d1 and d3 p.i. G. Fluorescent particle uptake by alveolar macrophages in vivo. For flow cytometric analysis, neutrophil-depleted mice were challenged with 1.5 × 10^7^ of flow particles. Percentage of flow particle positive (FP+) cells was assessed in AMs (CD45^+^Ly6G^−^CD11c^+^SiglecF^+^) from the wild-type and *Adipoq*^−/−^ mice by flow cytometry. Data are a summary of two independent experiments. *p < 0.05, **p < 0.01, ***p < 0.001.

Although APN pathway-deficient mice displayed increased IA severity, fungal burden, and an increased AM inflammatory phenotype, the mechanism of decreased fungal clearance remains unknown. To determine if APN-deficient AMs were deficient in fungal uptake and killing, we used dual viability/tracking fluorescent *Aspergillus* reporter FLARE conidia [18] to compare changes in conidia uptake and viability in wild-type and APN-/- AMs ex vivo and in vivo. In APN-deficient AMs cultured ex vivo, uptake and killing of dual-labeled FLARE conidia was decreased after 10 hours when observed by microscopy (**Fig 3A** and **S1** Video). When loss of *A. fumigatus* viability was compared by image analysis, killing of FLARE conidia by APN-deficient AMs was markedly decreased compared to wild-type AMs (**Fig 3B**). Uptake and killing were also reduced in APN-deficient AMs when analyzed by flow cytometry, even after filtration to remove hyphae that may reduce significance in flow cytometric vs microscopic analyses (**Fig 3C** and **3D**). Reduced killing by APN-deficient AMs was confirmed in vivo in FLARE-infected neutropenic mice, with a significant difference in conidial viability by day 3 post-infection (**Fig 3E** and **3F**), while uptake of 1 µm inert particles was not significantly different between wild-type and APN-deficient AMs in vivo (**Fig 3G**). Collectively, these results demonstrate that AM fungal uptake and killing are decreased in the absence of APN.

### The AdipoR agonist AdipoRon decreases inflammation and fungal burden in APN-deficient mice with IA.

**Fig 4:**
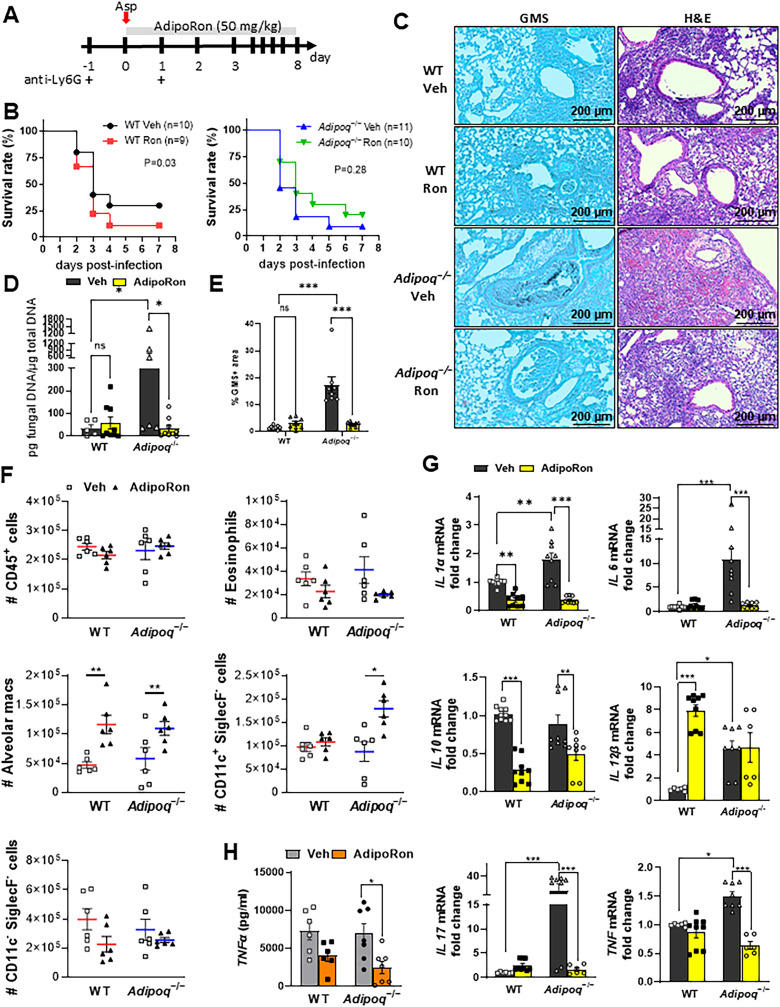
The AdipoR agonist AdipoRon decreases inflammation and fungal burden in APN-deficient mice with IA. Wild-type (C57BL/6), and *Adipoq*^−/−^ mice were neutrophil depleted and involuntarily aspirated *A. fumigatus* conidia as described in Materials and Methods. A. Time course for neutropenic mouse model of infection and AdipoRon treatment. B. Effect of AdipoRon on the survival rate of WT and *Adipoq*^−/−^ mice. C. Representative GMS and H&E lung sections. D. Fungal burden determined by quantitative PCR of fungal DNA from lung homogenates. E. Fungal burden determined by quantification of GMS staining. F. Total number of CD45^+^ cells, eosinophils, AMs, CD11c^+^SiglecF^−^, and CD11c^−^SiglecF^−^ cells isolated from the mice with IA as determined by flow cytometry. G. qRT-PCR analysis for mRNA expression of the indicated cytokines. H. TNF secretion in BALF quantified at the protein level by ELISA. Data are a summary of 2-3 independently performed experiments. *p < 0.05, **p < 0.01, ***p < 0.001.

A small molecule agonist (AdipoRon) that binds and activates signaling through both AdipoR1 and AdipoR2 improved insulin resistance and glucose intolerance and increased the lifespan of obese mice [19]. A role for AdipoRon in dampening inflammatory responses in human lung macrophages via inhibition of production of TNF, IL-6, and inflammatory chemokine production has also been reported [20]. However, the ability of AdipoRon therapy to improve IA outcomes remained unknown. We treated infected neutropenic wild-type or APN-deficient mice with AdipoRon by involuntary aspiration daily for up to eight days (**Fig 4A**). Although AdipoRon treatment in WT and APN-deficient mice did not significantly improve survival compared to vehicle treatment (**Fig 4B**), lung hyphal growth and fungal burden were markedly reduced in AdipoRon-treated APN-deficient mice compared to vehicle-treated APN-deficient mice at 3 days post-infection (Fig 4C-4E). A pathology score obtained by image quantification of H&E-stained sections indicated decreased inflammatory infiltration in the lungs of AdipoRon treated APN-deficient mice (**S4 Fig**). Airway eosinophil recruitment was reduced in AdipoRon treated APN-deficient mice, while AMs were increased in both AdipoRon-treated WT and APN-deficient mice **(Fig 4F**). Transcription of IL-1a, IL-6, IL-10, IL-17A, and TNF also decreased with AdipoRon treatment in APN-deficient lungs and decreases in IL-1a and IL-10 were also evident in wild-type mice (**Fig 4G**). In contrast, IL-12b transcription appeared to be increased by AdipoRon, but only in wild-type lungs. An AdipoRon-mediated decrease in TNF was also confirmed in APN-deficient BALF at the protein level (**Fig 4H**). These results indicate that APN pathway stimulation by AdipoRon decreases lung fungal burden and dampens lung inflammation in APN-deficient mice with IA.

### AdipoRon increases fungal killing and inhibits the inflammatory phenotype of APN-deficient AMs.

**Fig 5:**
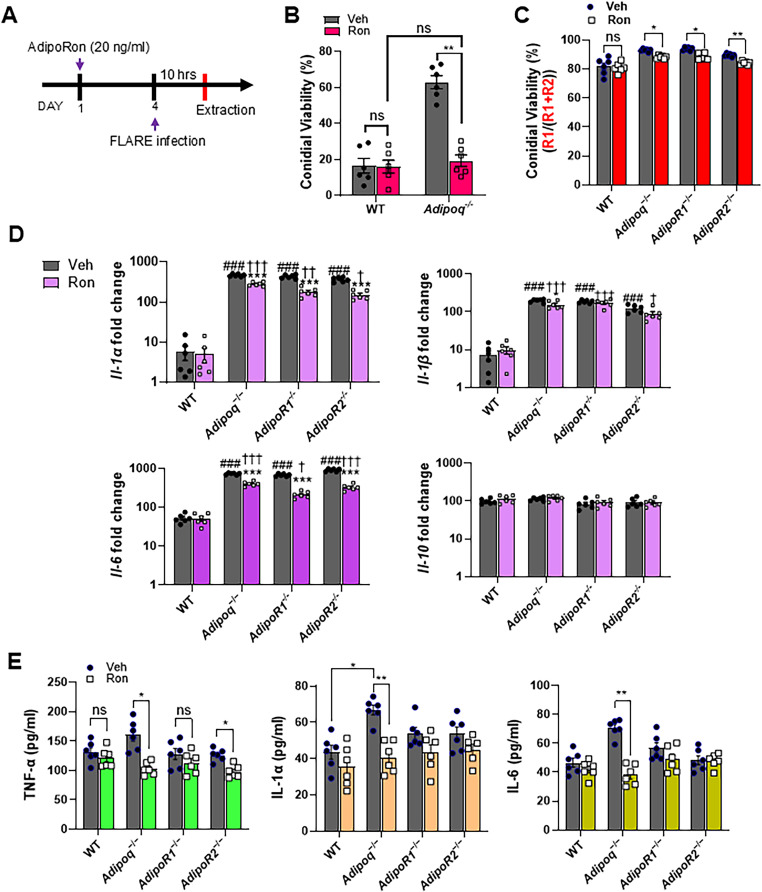
AdipoRon increases AdipoR-dependent AM fungal killing and reverses inflammatory phenotype. Wild-type or APN pathway-deficient AMs were infected and treated with AdipoRon or vehicle to determine the effect of AdipoRon on fungal killing, cytokine transcription and secretion as described in *Materials and Methods*. A. Timeline for *ex vivo* cultured AMs challenged with conidia with or without AdipoRon treatment. B. Microscopic image quantification of *Aspergillus* viability (R1/(R1 + R2)) in *ex vivo* cultured AMs by using FLARE conidia. The infection was done ex-vivo in 1:1 ratio (AMs: FLARE) for 15 hours. C. Flow cytometric quantification of *Aspergillus* viability. D. qRT-PCR analysis for mRNA expression of the indicated cytokines. Differences between experimental groups that resulted in a *p* value < 0.05 were considered significant. An asterisk (*) indicates a significant difference versus corresponding non-infected controls. A hash (#) indicates a significant difference between WT versus KO mice in vehicle groups. A dagger (†) indicates a significant difference between WT groups versus KO mice in AdipoRon-treated groups. E. TNF, IL-1a, and IL-6 secretion quantified at the protein level using *ex vivo* cultured AMs by ELISA. Data are a summary of two independently performed experiments. *p < 0.05, **p < 0.01, ***p < 0.001.

Although AdipoRon treatment influenced lung fungal growth and dampened inflammation in APN-/- mice with IA, the specific effect on AMs remained unknown. To determine the effect of AdipoRon on AM phenotype and fungal uptake and killing, we infected WT or APN pathway-deficient AMs with AF293 or FLARE conidia after 24 hours treatment with AdipoRon or control vehicle (containing DMSO), and harvested AMs ten hours later for microscopic, flow cytometric, qRT-PCR, and ELISA analyses (**Fig 5A**). When quantified by microscopy, conidia viability was decreased by AdipoRon treatment of APN-deficient but not wild-type AMs (**Fig 5B**). Conidia viability was also reduced in APN pathway-deficient AMs by AdipoRon when measured by flow cytometry after cell straining to remove hyphae (**Fig 5C**). Inflammatory cytokine transcription of IL-1 *α* , IL-1β, and IL-6 were all decreased by AdipoRon treatment in APN-deficient AMs, while the anti-inflammatory cytokine IL-10 was not affected by AdipoRon regardless of APN sufficiency (**Fig 5D**). Secretion of TNF, IL-1α and IL-6 protein were also significantly decreased in AdipoRon-treated APN-deficient AMs (**Fig 5E**). These results indicate that AdipoRon treatment acts on APN-deficient AMs to improve fungal killing and inhibit an inflammatory phenotype.

### AdipoRon-enhanced killing of A. fumigatus requires either AdipoR1 or AdipoR2.

**Fig 6:**
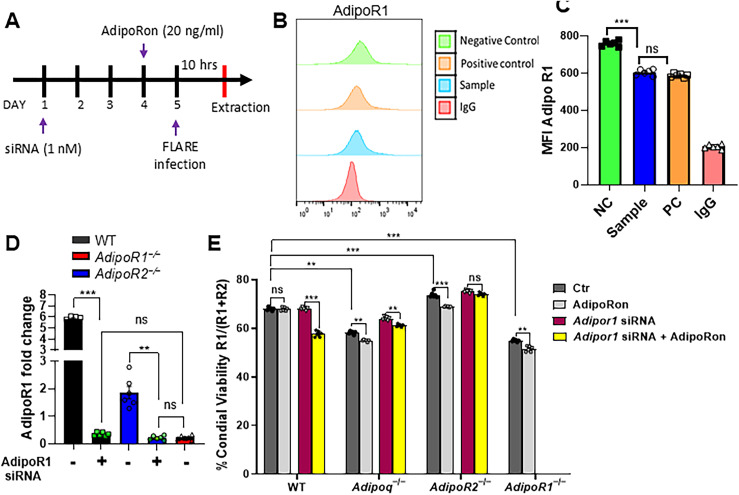
AdipoRon-enhanced killing of *A. fumigatus* requires either AdipoR1 or AdipoR2. AMs from Wild-type or APN pathway deficient AMs were treated with AdipoR1 siRNA and/or AdipoRon, then infected with FLARE conidia to determine the effect on and requirement for AdipoR expression in stimulation of fungal killing as described in *Materials and Methods*. A. Timeline for primary AM cultures treated with *AdipoR1* siRNA, details in *Materials and Methods* section. B. Histogram graphs obtained from flow cytometry analysis at 72 hours post siRNA treatment. The positive control represents the ideal *Adipor1* knockdown control, while the negative control is in the absence of functional siRNA. C. MFI AdipoR1 is graphed from negative control, sample and positive control from flow cytometric analysis. D. qRT-PCR analysis for mRNA expression of the *Adipor1* in AMs from the APN-deficient mice with siRNA treatment. E. Conidial viability (R1/(R1 + R2)) in *ex vivo* cultured AMs with siRNA and/or AdipoRon by flow cytometry. Data are a summary of two independently performed experiments. **p < 0.01 and ***p < 0.001.

We observed that decreased AM transcription of *Adipor1* and *Adipor2* by infection was partially restored by AdipoRon treatment (S2E). Since AdipoRon enhances AM-mediated fungal killing, we aimed to confirm the requirement of AdipoR1 and AdipoR2 by using siRNA to target expression of AdipoR1 in wild-type, APN-, and AdipoR2-deficient AMs (**Fig 6A**). Knockdown of AdipoR1 was confirmed by flow cytometry to reduce expression to identical levels observed in AMs from AdipoR1-deficient mice (Fig 6B-D). Moreover, fungal killing was improved by AdipoRon treatment in wild-type, APN-, AdipoR1- (knockout mice and siRNA), and AdipoR2-deficient AMs, but was not improved with siRNA-targeted depletion of AdipoR1 in AdipoR2-deficient AMs (**Fig 6E**). These results show that either canonical APN receptor is critical for AdipoRon-mediated enhancement of AM fungal killing.

### Adiponectin promotes LC3-associated phagocytosis of A. fumigatus conidia.

To confirm the AdipoRon-mediated phenotypic shift in AMs, we compared AdipoRon treatment with vehicle in infected and uninfected APN-deficient AMs by global RNAseq analysis. In uninfected AMs, more genes were upregulated in response to AdipoRon treatment, while the opposite was observed in AMs after 12 hours post-infection, with AdipoRon treatment associated with gene silencing (**S3A Fig**). Inflammatory -associated gene expression was suppressed by AdipoRon treatment in infected AMs, including *Il1a*, *Il1b*, *Ccl2*, *Cd38*, *Tlr4*, and *Hif1a*, while the anti-inflammatory *Tgfb* was increased, as were autophagy genes, including *Map1lc3a* and *Map1lc3b* genes that are critical for LAP (LC3-associated phagocytosis) (**S3B Fig**). In contrast, inflammatory gene expression appeared to be increased by AdipoRon in uninfected AMs. GSEA pathway analysis confirmed inhibition of cytokine signaling by AdipoRon in infected AMs and identified activation of gene pathways associated with oxidative phosphorylation (**S3C Fig**), a metabolic pathway favored by alternatively activated macrophages [21]. Our RNAseq results thus confirm an AdipoRon-mediated inhibition of the inflammatory phenotype of APN-deficient AMs in response to fungal infection and identify a potential role for autophagy induction.

**Fig 7:**
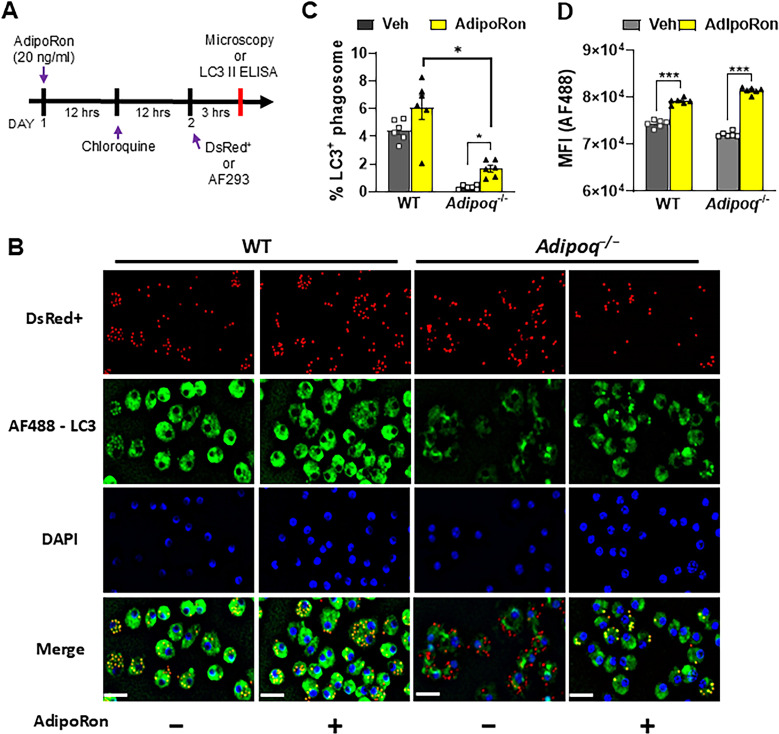
Adiponectin promotes LC3-associated phagocytosis of *A. fumigatus* conidia. Wild-type or *Adipoq*-/- AMs were treated or vehicle-treated and infected with AF293 or DS-Red+ conidia and stained for LC3 and DAPI to determine the effect of AdipoRon on AM phagosomal LC3 expression. A. Timeline for LC3-associated phagocytosis analysis. B. Microscopy of LAP-dsRed conidia and LC3^+^ AMs at a magnification of 20X in *ex vivo* cultured AMs from infected WT and *Adipoq*^−/−^ mice with or without AdipoRon. The images from DsRed, FITC, DAPI channels are presented followed by the merged image. Scale bar: 35µm. C. Microscopic quantification of LC3^+^ phagosome (%) in *ex vivo* cultured AMs from infected WT and *Adipoq*^−/−^ mice with or without AdipoRon. D. The mean fluorescent intensity (MFI) obtained by flow cytometry indicates the expression level of LC3-AF488 in *ex vivo* cultured AMs from infected WT and *Adipoq*^−/−^ mice with or without AdipoRon. Data are a summary of two independently performed experiments. *p < 0.05 and ***p < 0.001.

Since LAP is an important mechanism of killing of *A. fumigatus* by macrophages [3], we aimed to determine if phagosomal LC3 activation is increased in APN-deficient AMs by AdipoRon treatment. Using immunofluorescence microscopy staining of LC3 in chloroquine-treated cells (**Figs S6A-B** and 7A), we observed clear colocalization of LC3 staining and DS-RED+ (viable) conidia in AdipoRon-treated, infected APN-deficient AMs (**Fig 7B**), with visibly less colocalization in untreated AMs that was confirmed by image quantification (Fig 7C). The median fluorescence intensity (MFI) of LC3+ macrophage staining was also increased by AdipoRon in both wild-type and APN-deficient AMs as measured by flow cytometry (**Fig 7D**) and increased in infected vs uninfected AMs (**S6C-D Fig**). Increased conversion of LC3-I to active LC3-II protein in AdipoRon-treated APN-deficient AMs was confirmed by ELISA (**S6E Fig**). Collectively, these results demonstrate a role for adiponectin in LAP in AMs in response to *A. fumigatus* conidia.

## Discussion

To our knowledge, our study is the first to identify a role for adiponectin in LC3-associated phagocytosis (LAP) in response to infection. Previous studies reported roles for adiponectin in LC3-mediated autophagy in skeletal muscle cells [22,23], cardiomyocytes [24], or macrophages in a mouse model of atherosclerosis [25]. In addition, AM autophagy is critical for prevention of spontaneous lung inflammation driven by airway microflora [26]. Although others have identified a role for the LC3 pathway in efferocytosis by macrophages [27], and although adiponectin promotes macrophage efferocytosis [28,29], a direct connection between adiponectin, LC3/autophagy and macrophage efferocytosis is lacking. Since macrophage autophagy and removal of apoptotic cells are important mechanisms to limit lung inflammatory pathology [26,29,30], it will be of interest in future studies to determine their role in adiponectin-mediated dampening of inflammation in IA.

A number of studies have defined a critical role for LAP in protection from *A. fumigatus* infection [3,31–33]. LAP dampens inflammation in response to *A. fumigatus* by inhibition of the NLRP3 inflammasome and IL-1b via activation of DAPK1 [31]. In our study, we observed that AdipoRon decreased transcription of IL-1α and IL-1β genes in APN-deficient AMs. Interestingly, blockade of IL-1 receptor signaling (by IL-1ra) restored autophagy/LC3 recruitment in NADPH oxidase-deficient macrophages, a hallmark myeloid cell deficiency of Chronic Granulomatous Disease [34]. Since both AdipoRon and IL-1ra promote LC3 activation, it will be important in future studies to determine if AdipoRon could restore autophagy in NADPH oxidase-deficient macrophages, and to identify shared regulatory pathways.

A previous study reported that LAP-associated fungal clearance was inhibited by fungal melanin, a virulence factor of *A. fumigatus* [32]. How APN stimulates LAP in response to *A. fumigatus* and the ability to overcome LAP interference by fungal melanin remain unknown. Furthermore, we observed that chitin inhalation resulted in decreased production of lung adiponectin and AdipoR1 expression in murine airway cells [35]. It will be of interest to determine the effect of AdipoRon on recognition of multiple fungal pathogen-associated molecular patterns by WT and APN pathway-deficient AMs, specifically in the context of LC3 activation.

Adiponectin has two canonical signaling receptors with broad, yet different cell/tissue distribution [36]. We observed that both AdipoR1 and AdipoR2 contribute to adiponectin-mediated protection from IA and dampening of inflammatory pathology. However, some differences were evident, as AdipoR1-deficient mice with IA exhibited the highest levels of lung inflammatory cytokine transcription that was not as clear in AdipoR1-deficient AMs, suggesting that immune pathology in AdipoR1-deficient mice was not driven solely by the lack of AdipoR1 signaling in AMs, but also by other immune and resident lung cells, with a contribution of potentially enhanced signaling through AdipoR2. AMs express both AdipoRs, and their expression was modulated by *A fumigatus* infection, most significantly for AdipoR1, which was increased in response to infection in WT AMs but decreased in APN-deficient AMs. Furthermore, both AdipoR1 and AdipoR2 were required for AdipoRon-mediated improvement in *A. fumigatus* killing, as AdipoRon was not effective in AdipoR1/R2-deficient AMs, confirming a role for both receptors in APN-mediated enhancement of AM function. However, the relative contribution of AdipoR-expressing AMs in adiponectin-mediated protection from infection remains unclear and thus remains a subject of future investigation.

Although AMs are likely contributors to fungal clearance, inflammatory pathology mediated by other cells may also play a distinct role in our model, as AdipoRon therapy of APN-deficient mice with IA improved fungal clearance without significantly improving survival. Previous work by others has highlighted the potential role of inflammatory pathology in *A. fumigatus* infection [2], and a previous study of a model of *Candida* infection provides further evidence that decreases in fungal burden are not invariably linked to improved survival [37]. Thus, while AdipoRon enhances antifungal immune responses in AMs, it may not fully compensate for the loss of APN’s broader immunoregulatory effects, particularly in the setting of severe infection and neutropenia.

AMs are dynamic cells, and their activation and polarization falls on a spectrum from M1 to M2, as these binary designations are not indicative of a static phenotype [38,39]. In our study, AM treatment with AdipoRon decreased expression of inflammatory/M1-like genes and activated alternative/M2-like genes in *A. fumigatus*-infected APN-deficient AMs, although these phenotypes are not identical to previously published studies using classical activation stimuli, such as LPS/IFNg for M1 and IL-4 for M2 [40,41]. It is thus more appropriate to refer to APN-deficient AMs as ‘M1-like’ and AdipoRon-treated AMs as ‘M2-like’ cells [42,43]. Perhaps more importantly, AdipoRon treatment of APN-deficient AMs resulted in activation of genes associated with oxidative phosphorylation that is associated with M2-like macrophages, in contrast to glycolysis-favoring M1-like macrophages [21]. In support of increased oxidative metabolism for AdipoRon-treated APN-/- AMs, arginase-2 (*Arg2*) expression was also increased, and mitochondrial *Arg2* was recently identified as critical for induction of oxidative phosphorylation in inflammatory macrophages [44]. Collectively, the results of our study confirm a role for APN in skewing AMs toward an alternative/M2-like phenotype.

Obesity is widely accepted as an inflammatory disease characterized by adipose tissue inflammation and decreased APN and APN receptor expression [6,45–48], yet the effect of decreased activation of the APN pathway in response to *A. fumigatus* infection is not known. In response to infection with *Listeria monocytogenes*, both obese and APN-deficient mice exhibited attenuated clearance that was characterized by an inflammatory phenotype in bone marrow macrophages that was partially restored by AdipoRon [7]. Furthermore, obesity is associated with an increased inflammatory phenotype in alveolar macrophages [49]. It is therefore possible that stimulation of the APN pathway with AdipoRon will improve AM killing of *A. fumigatus* in obese mice, an outcome that would support the APN pathway as a potential therapeutic route in IA patients with low APN levels.

## Materials and Methods (See also Key Resources (S1 Table) and Supporting Methods (S1 Text).

### Ethics Statement

All animal handling and experimental procedures were performed in accordance with the recommendations found in the Guide for the Care and Use of Laboratory Animals of the National Institutes of Health. The work in this study was approved by the Institutional Animal Care and Use Committee of the host campus of Indiana University School of Medicine-Terre Haute, Indiana State University (Protocol #2056115-1).

### Mice

C57BL/6 and adiponectin-deficient (*Adipoq*−/−) and *AdipoR2*+/− mice were obtained from The Jackson Laboratory, while *AdipoR1+/-* mice were obtained from the Mutant Mouse Resource and Research Center (MMRRC). To obtain homozygous deletion mice for *AdipoR1*−/−, and *AdipoR2*−/− mice, heterozygous mice were breed with F1 animals heterozygous and offspring were screened by PCR, with loss of protein confirmed in a subset of animals by flow cytometry.

### Fungal strains and cultivation

The clinical isolate AF293 was previously obtained from the Fungal Genetics Stock Center and grown on Malt Extract Agar (MEA) plates at 22˚C. To generate FLARE conidia, AF293-dsRed conidia (provided by Dr. Tobias Hohl, Memorial Sloan-Kettering) were rotated in 0.5 mg/ml Biotin XX, SSE in 1 ml of 50 mM NaHCO_3_ for 2 h at 4˚C, washed with 0.1 M Tris-HCl (pH 8.0), incubated with 0.02 mg/ml Af633-streptavidin for 30 min at RT, and resuspended in PBS for use [50].

### Fixing and Swelling Conidia

14-day-old AF293 grown on MEA plates were used. Two ml of extraction beads were swirled gently 5 times. The beads were taken into a 15 ml centrifuge tube and 5 ml of 1X DPBS was added to this tube. The sample was vortexed thoroughly. The conidia in the DPBS was then counted using a hemocytometer. The DPBS with the conidia was transferred to a fresh 15 ml centrifuged tube and was centrifuged for 10 minutes at 10,000 rpm. The supernatant was discarded and 5 to 6 ml of RPMI-1640 was added and incubated at 26˚C for 2 to 5 hours. The tube was then centrifuged for 10 minutes at 10,000 rpm and the supernatant was discarded. Fixation buffer was added and was left overnight at 4˚C. 0.1 M NH4Cl was added and vortexed thoroughly. The sample was then centrifuged at 10,000 rpm for 10 minutes. After 3 times 1X D DPBS was added to adjust the concentration according to the infection concentrations.

### Fungal aspiration and infection

Neutrophils were depleted by i.p. injection of 0.5 mg of α-Ly6G (1A8 clone) 24 h pre- and post-infection for invasive pulmonary aspergillosis mice model, as described [8]. Isoflurane-anesthetized mice involuntarily aspirated 50 μl of suspension including 1 – 2 × 10^7^ of conidia. For survival, infected mice were monitored for 8 days post-infection. For further analyses, a subset of infected mice were euthanized with sodium pentobarbital and mouse BAL cells and lungs were harvested 24 h or 72 h later for further analyses.

### Histology

At 3 days post-infection, lungs were harvested after perfusion with 5 ml of DPBS followed with 5 ml of 10% phosphate buffered formalin and inflated with 15 ml of formalin in 50 ml tubes. Lung tissue was embedded in parafilm after dehydration and sectioned in 4 μm slices using xylene and ethanol and stained with modified Gomori’s modified methanamine silver (GMS) and hematoxylin and eosin (H&E) staining [8]. Briefly, the sample on the slide was deparaffinized with xylene and ethanol and rehydrated in periodic acid solution for 5 to 10 min in 62°C. The slide was rinsed with deionized water 6 times and reacted with pre-warmed silver methenamine solution for 30 min in 62°C. After washing with water, the slide was reacted with gold chloride solution followed by sodium thiosulfate solution. After washing with water, the slide was counter stained with 5% fast green solution. After dehydration in ethanol and xylene, the stained sample was mounted by using permount solution. Quantification of GMS staining to determine fungal burden was performed as previously described [51].

### Quantitative PCR fungal burden assay

At 72 h post infection, lungs were harvested, frozen in liquid nitrogen and lyophilized. Genomic DNA was extracted from homogenized lung tissue with DNA extraction buffer for *Aspergillus* nucleic acids and subsequent phenol/chloroform extraction. Quantitative PCR (qPCR) fungal burden assays were performed using 0.2 μg of genomic DNA with 18S rRNA-encoding DNA primers and probe sets with a modified probe quencher (50-/56-FAM/AGC CAG CGG/ZEN/CCC GCAAAT G/3IABkFQ/-30) [52]. Quantitative PCR was performed using the CFX Connect Real-Time System. Threshold values were used to calculate the corresponding fungal DNA content in the lung tissues.

### Tissue RNA extraction and gene expression analysis

The lungs were lyophilized overnight. Total RNA was extracted from homogenized lungs in TRIzol reagent. Following the upper phase, further RNA purification was performed using a Qiagen RNeasy column and DNase treatment per the manufacturer’s recommendations [53]. According to the manufacturer’s protocol, cDNA was synthesized using High-Capacity cDNA Reverse Transcription Kit (Thermo Fisher Scientific). Mouse β-actin was used as the housekeeping gene for cytokine analysis in the murine model. Most primers were designed using the Primer3 software (version 0.4.0) from the whole sequence available in GenBank. PowerUp SYBR Green Master Mix was used to perform real-time quantitative PCR.

### BALF flow cytometric analysis

BALF cell composition was determined by flow cytometric analysis with staining of surface markers. In brief, BALF was centrifuged, and the cell pellet was washed with 1 ml of FACS buffer (PBS, 5% FBS, 50 mM EDTA). The washed pellet was lysed of red blood cells with ACK lysis buffer and stained with 10% rat serum, Fc receptor blocking Ab, and antibodies: rat anti-mouse Ly6G-FITC or -BV785, rat anti-mouse SiglecF-PE or -Superbright600, rat anti-mouse CD45-PerCP, and rate anti-mouse CD11c-PE-Cy7 or -SparkBlue550 [53,54]. For staining of AdipoR1, the washed pellet was resuspended in blocking solution containing 10% donkey serum, Fc receptor blocking Ab, and 1% Bovine serum albumin in PBS for 30 min, incubated with the primary AdipoR1 antibody for 1 h and incubated with the IgG secondary antibodies [35]. Analyses were performed by flow cytometric analysis on a Luminex/Guava EasyCyte HT system or a Luminex/Cytek Aurora. Flow cytometric data were analyzed using FlowJo software.

### BALF ELISA analysis

Serum and BALF supernatants were collected after centrifugation. ELISA analyses were performed for TNFα cytokine (PeproTech) and Adiponectin (R&D Systems) as described by the manufacturer’s protocol. The range for ELISA is 8 to 1000 pg/ml and 3.9 to 250 ng/ml, respectively.

### Alveolar macrophage extraction and culture

Seven- to 8-week-old mice were euthanized, and an 18-gauge catheter was inserted into the trachea and alveolar macrophages were harvested from mouse lungs with sterile filtered PBS containing 2 mM EDTA (diluted 1:250 from 0.5 M EDTA stock solution) and 0.5% Fetal bovine serum (FBS) in 5ml BAL fluid. Cells were separated from the BALF by centrifugation at 400 × *g* for 6 min at 4°C and the cells were suspended in RPMI 1640 supplemented with penicillin (100 U/ml), streptomycin (100 U/ml), 5% heat-inactivated FBS, 20 ng/ml GM-CSF and 143mM beta-mercaptoethanol. BALF cells were plated in Corning Costar Flat Bottom 12 well Cell Culture Plates at a concentration of 5 × 10^5^ cells per well. The cells were allowed to adhere for 24 hours at 37°C under a humidified atmosphere with 5% CO_2_ and were washed with PBS thrice and cell culture media was added, allowing only the adherent AMs to culture in the wells. The cell culture media was changed on day 3, day 6 and day 9 of the cultures. AMs were fully confluent and ready for treatment and infection on day 10. The wells for divided into DMSO control (vehicle) and AdipoRon treatment group with AF293/FLARE infection.

### Quantitative RT-PCR for ex-vivo gene expression analysis

AMs were treated with 20 uM AdipoRon or vehicle-treated for 24 hours, followed by infection with AF293 fixed and swollen conidia using RPMI 1640 medium at a concentration of 9 conidia per cell for RT-PCR. After 10 hours of infection, cells were ready for RNA extraction and cDNA synthesis. RNA extraction from AMs was done using Qiagen RNeasy Mini kit, following the manufacturer’s protocol. According to the manufacturer’s protocol, cDNA was synthesized using High-Capacity cDNA Reverse Transcription Kit (Thermo Fisher Scientific). PowerUp SYBR Green Master Mix was used to perform real-time quantitative PCR.

### AM supernatant ELISA

After culturing for 10 days, AMs were grouped into uninfected, vehicle (DMSO), infected and AdipoRon-treated groups. After the infection at a concentration of 9 conidia per cell with fixed and swollen AF293 conidia for 10 hours, the supernatants were collected and analyzed for TNF, IL-1a, and IL-6 expression.

### Flow cytometric analysis for FLARE uptake and killing in AMs

Conidia was harvested from 7-10 days old Minimal Media (GMM) plates. AF293-dsRed conidia were rotated in 0.5 mg/ml Biotin XX, SSE in 1 ml of 50 mM NaHCO3 for 2 h at 4˚C, washed with 0.1 M Tris-HCl (pH 8.0), incubated with 0.02 mg/ml Af633-streptavidin for 30 min at RT, and resuspended in PBS for use. For in-vitro conidial uptake and killing assays, 5x10^5^ FLARE conidia were added to 5x10^5^ AMs (1:1). After 10 hours AMs were harvested using cell scraper with slight force from the wells in 500 ul ice cold FACS buffer per well and centrifuged at 400 × *g* for 6 min at 4°C. The cell pellet was washed with 1 ml of FACS buffer (PBS, 5% FBS, 50 mM EDTA) and the samples were passed through 70µm filter to clear hyphae and have clear sample. The cells were analyzed by the flow cytometry for DsRed (Ex/Em: 558nm/583nm) and AF633 (Ex/Em: 631nm/650nm) fluorescence followed by gating using FlowJo.

### Inflammatory/alternative AM phenotyping

For AM phenotype analysis cells were stained with 10% rat serum, Fc receptor blocking Ab and antibody: *CD38 Monoclonal Antibody (90), APC-eFluor 780, eBioscience* for 15 min at room temperature. For staining with the antibody: *EGR2 Monoclonal Antibody (erongr2), PE-Cyanine7, eBioscience* cells were permeabilized using Cytoperm and processed for staining.

### Microscopic analysis for FLARE uptake and killing in AMs

AMs were plated in Cellvis 35 mm Glass bottom dish with 14 mm micro-well and were infected with FLARE/DsRed at a 1:1 ratio. Cells were live imaged after 6 hours on a ZEISS LSM780 confocal microscopy.

### LC3 phagocytosis assay

On day 10, cells were treated with 20 µM AdipoRon for 24 hours followed by treatment with 90µm chloroquine for 13 hours. Following chloroquine diphosphate treatment, lysosomal pH increases and the normal autophagic flux is disrupted, resulting in autophagosome accumulation. Cells were then infected with DsRed+ conidia for 120 min and then fixed with 3.7% formaldehyde in PBS for 15 min at room temperature. LC3B antibody kit for autophagy was used and the manufacturer’s protocol is followed for LC3 staining. Invitrogen Goat anti-Rabbit IgG (H+L) Cross-Adsorbed Secondary, Antibody, Alexa Fluor 488 was used for secondary staining. Cells were washed and counter stained with DAPI using VECTASHIELD Antifade Mounting Medium has been done. Microscopy was performed on OLYMPUS IX83 and analyzed as follows: nucleus: DAPI, LC3b: FITC, conidia: Cy5 channel at 20X.

### siRNA knockdown of *Adipor1*

TriFECTa RNAi Kit was used with Lipofectamine 2000 reagent for transfection. Adherent 10-day-old alveolar macrophages were treated with diluted AdipoR1 SiRNA in Lipofectamine (1:1 ratio) at 1nM per 5 x 10^5^ cells concentration for 72 hours. The cells were then quantified for AdipoR1 receptor expression using AdipoR1 RT-PCR and compared with provided positive and negative controls.

### Statistical analysis

Prism software was used for generation of figures and for statistical analyses (GraphPad). Unpaired *t* tests or ANOVA tests were used to measure statistical significance. Differences between experimental groups that resulted in a *p* value < 0.05 were considered significant.

## Supporting information

S1 Fig Inflammatory phenotype of APN-/- mice from a second strain. Wild-type (C57BL/6) and *Adipoq*^−/−^ mice (second strain obtained from Dr. Philipp Scherer) were neutrophil depleted and involuntarily aspirated 1 – 1.5 × 10^7^ of conidia as described in *Materials and Methods*. A. Survival rate. B. Representative GMS and H&E lung sections. C. Fungal burden determined by quantitative PCR of fungal DNA from lung homogenates. D. Fungal burden determined by quantification of GMS staining. E. Total number of CD45^+^ cells, eosinophils, AMs, CD11c^+^SiglecF^−^, and CD11c^−^SiglecF^−^ cells isolated from the mice with IA as determined by flow cytometry. F. qRT-PCR analysis for mRNA expression of the indicated cytokines. G. qRT-PCR analysis for mRNA expression of *Adipor1* and *Adipor2*. H. TNFα secretion in BALF quantified at the protein level by ELISA. I. Representative flow cytometric dot plots with gating. Data are a summary of two independently performed experiments. *p < 0.05, ***p < 0.001.
(TIF)

S2 Fig AdipoRs expression in the Lung and BALF in APN pathway-deficient mice with invasive aspergillosis. Mice were infected with *A. fumigatus* or left uninfected and BALF or lungs were harvested for ex vivo stimulation or quantification of Adipoq or AdipoR expression by qRT[-PCR or flow cytometry. A. Expression of *Adipoq* and *Adipor* genes in lung homogenates and BALF cells from non-infected (NI) and conidia-infected WT mice. Lung and BALF were collected from the mice at 3 dpi. B. Summary of median fluorescence intensities of AdipoR1 staining on AMs, eosinophils, CD11c^+^SiglecF^−^, and CD11c^−^SiglecF^−^ cells from non-infected (NI) and conidia-infected WT mice. C. qRT-PCR analysis for mRNA expression of *Adipor1* and *Adipor2* in lung homogenates. Wild-type (C57BL/6), *Adipoq*^−/−^, *AdipoR1*^−/−^, and *AdipoR2*^−/−^mice were neutrophil depleted and involuntarily aspirated *A. fumigatus* conidia. D. Flow cytometry staining of AdipoR1. Frequency of AdipoR1+ in ex-vivo cultured AMs in WT and *Adipoq*^−/−^ mice. The histogram represents the AdipoR1 peak relative to IgG control in infected vs uninfected. E. qRT-PCR analysis for mRNA expression of *Adipor1* and *Adipor2* from the ex-vivo cultured AMs. The AMs were challenged with AF293 conidia with or without AdipoRon treatment. Data are a summary of two independently performed experiments. *p < 0.05, **p < 0.01, ***p < 0.001.
(TIF)

S3 Fig Gene expression in AdipoRon treated/untreated and infected/ uninfected *Adipoq−/−* AMs by RNAseq analysis. AMs were infected with swollen AF293 conidia with 1:9 cells/conidia for 10 hours, or left uninfected, with or without AdipoRon treatment, followed by RNA extraction for RNAseq analysis. A. Volcano plot depicting relative changes in gene expression of AdipoRon-treatment in APN-deficient AMs, infected (top) or uninfected (bottom). B. Heat map representation of the genes with highest differential expression in infected (left) and uninfected (right) APN-deficient AMs. C. GSEA-GO analysis of gene pathways that are both differentially expressed by AdipoRon treatment in infected (left) and uninfected (right) APN-deficient AMs.
(TIF)

S4 Fig Evaluation of lung histopathology in mice with IA after treatment with vehicle or AdipoRon . Lung tissues were harvested 3 days post-infection with conidia from vehicle or AdipoRon-treated mice. Histological sections of lungs were stained with H&E. Results were summarized as the average of the combined scores for the distinct parameters evaluated.
(TIF)

S5 Fig Evaluation of AM phenotype . AMs were grown as described in *Materials and Methods* and stained for Siglec-F, CD11c, F4/80 and CD11b or tested by qRT-PCR for expression of AM-associated genes. A. Representative flow cytometric dot plots. B. % cells Siglec-F+CD11c+ and F4/80+CD11b- was calculated from the data in S5A Fig. C. On day 15 of alveolar macrophage culture, the RNA was extracted followed by qRT-PCR analysis for the AMs markers: *Pparγ* and *Car4*. The relative fold change was calculated.
(TIF)

S6 Fig Effects of Chloroquine, A. fumigatus infection, and AdipoRon treatment on LC3 expression in WT and *Adipoq-/-* AMs . AMs were cultured in ex vivo from WT, without and with Chloroquine, infection, or AdipoRon treatment. A. Microscopy of LAP-dsRed conidia and LC3+ AMs at a magnification of 20X. The images from DsRed, FITC, DAPI channels are presented followed by the merged image. Scale bar: 35µm. B. Microscopic quantification of % co-localization of LC3+ phagosome and DsRed+ conidia in *ex vivo* cultured AMs from WT infected group, without and with chloroquine treatment. C. Microscopy of LAP-AF293 conidia and LC3+ AMs at a magnification of 20X in *ex vivo* cultured AMs from WT uninfected group and WT infected group. The images from FITC, DAPI channels are presented followed by the merged image. Scale bar: 35µm. D. Mean Fluorescent Intensity of LC3 obtained from S6C Fig is graphed indicating the expression of LC3 without and with AF293 infection. E. LC3-II is quantified at the protein level using the ex-vivo cultured AMs by ELISA from cell lysates. Data are a summary of two independently performed experiments. *p < 0.05, **p < 0.01.d
(TIF)

S1 Video Time course of uptake and killing in WT and APN-deficient AMs. AMs were plated and infected as mentioned in the methods. Cells were live imaged by confocal microscopy for T= 8min under 40X water immersion lens. The black arrows indicate the live conidia at T=0 min and the white arrows indicate the conidia at T=8min.
(MP4)

S1 Table Key Resources. Resources and reagents used in this study.
(DOCX)

S1 Data Minimal Data Set. All data tables have been exported into the supporting pdf file, with tables appearing in the order they are presented.
(PDF)

S1 Text Supporting Methods. Methods for supporting data figures.
(DOCX)
