# Supplementary figures and images for: Adiponectin pathway activation dampens inflammation and enhances alveolar macrophage fungal killing via LC3-associated phagocytosis
Source: PLoS Pathog. 2025 Mar 17;21(3):e1012363. doi: 10.1371/journal.ppat.1012363 (PMC11949351; doi:10.1371/journal.ppat.1012363)

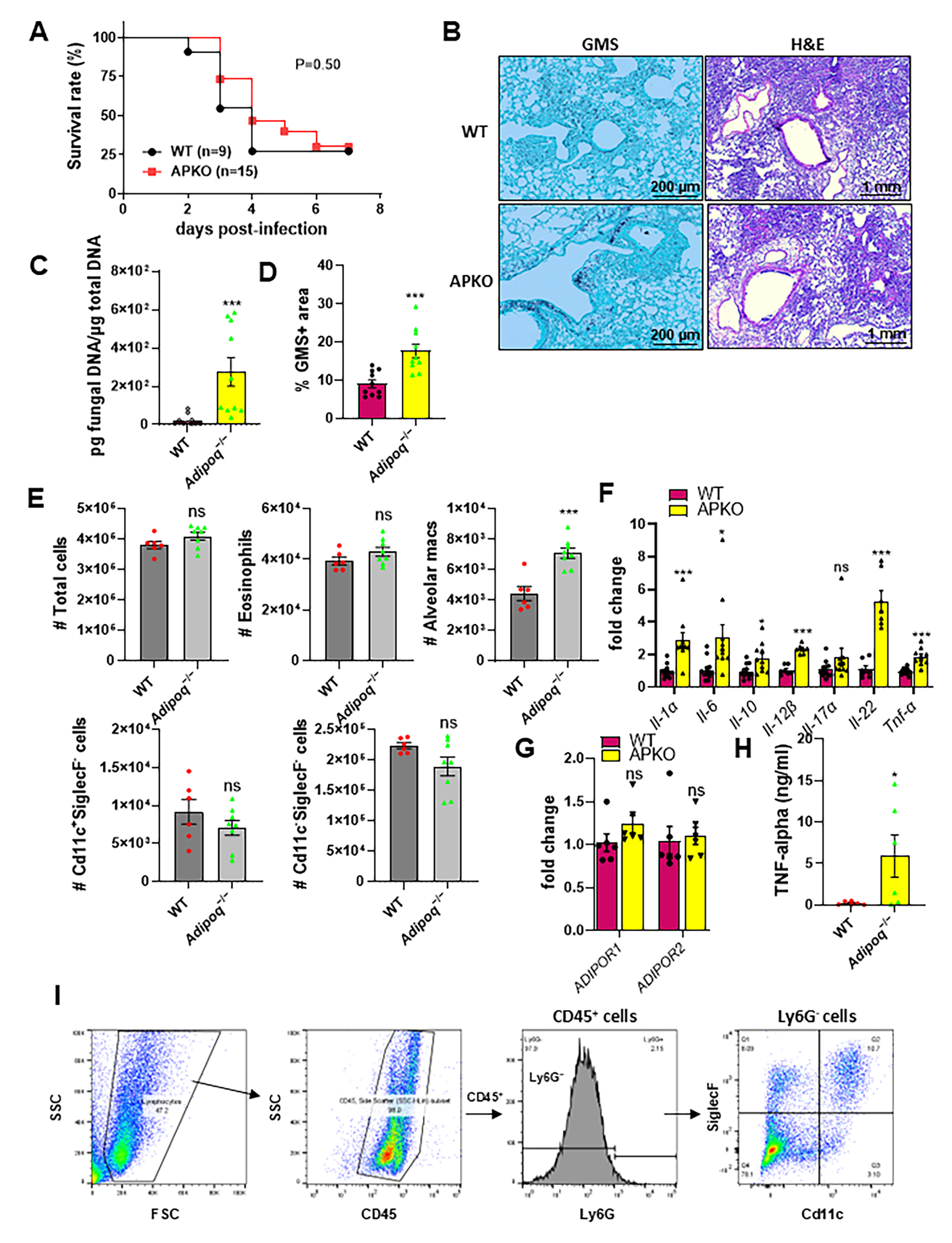

Supplement: S1 Fig — Wild-type (C57BL/6) and Adipoq−/− mice (second strain obtained from Dr. Philipp Scherer) were neutrophil depleted and involuntarily aspirated 1 – 1.5 × 10^7^ of conidia as described in Materials and Methods. A. Survival rate. B. Representative GMS and H&E lung sections. C. Fungal burden determined by quantitative PCR of fungal DNA from lung homogenates. D. Fungal burden determined by quantification of GMS staining. E. Total number of CD45+ cells, eosinophils, AMs, CD11c+SiglecF−, and CD11c−SiglecF− cells isolated from the mice with IA as determined by flow cytometry. F. qRT-PCR analysis for mRNA expression of the indicated cytokines. G. qRT-PCR analysis for mRNA expression of Adipor1 and Adipor2. H. TNFα secretion in BALF quantified at the protein level by ELISA. I. Representative flow cytometric dot plots with gating. Data are a summary of two independently performed experiments. *p < 0.05, ***p < 0.001. (TIF) [file ppat.1012363.s001.tif]

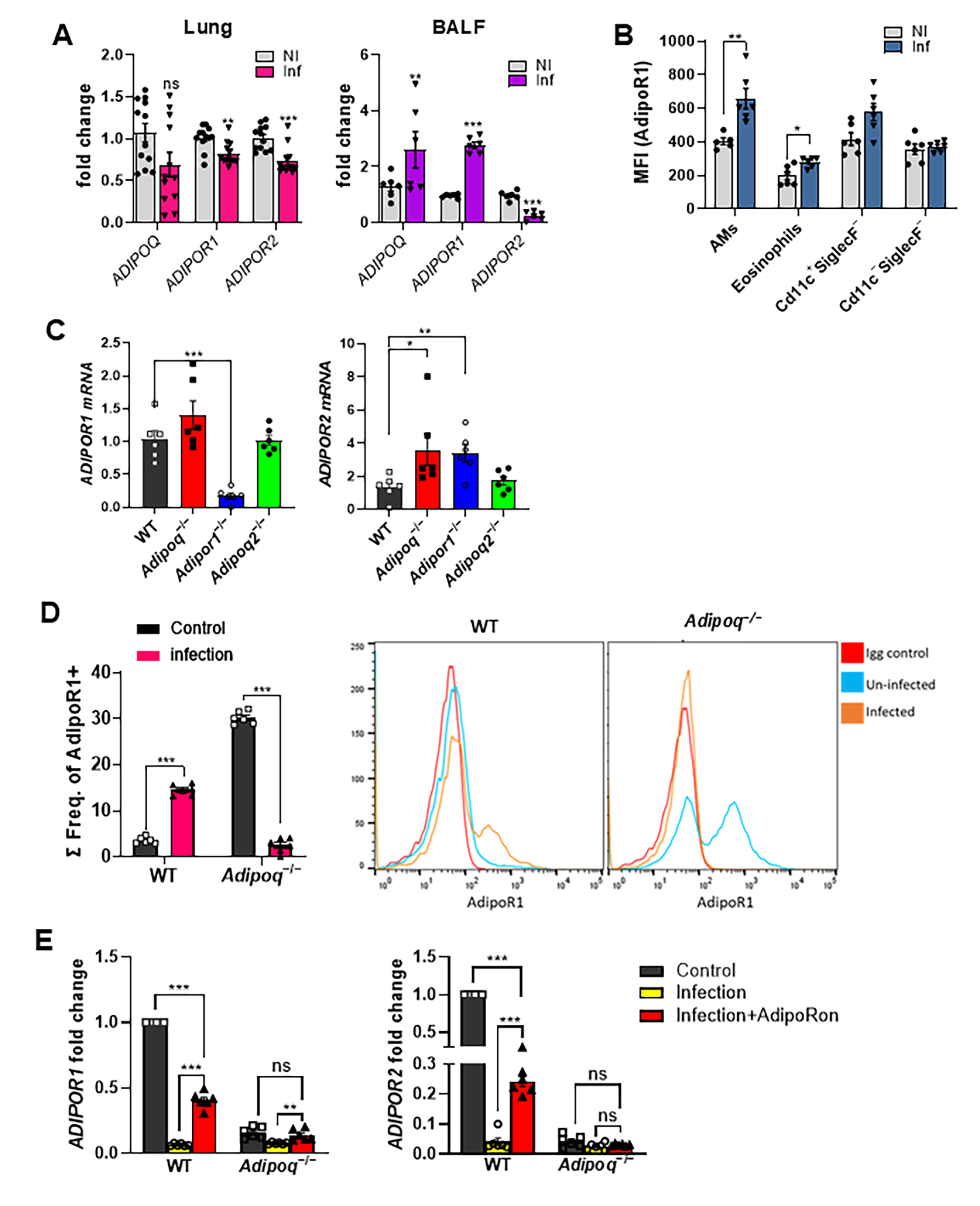

Supplement: S2 Fig — Mice were infected with A. fumigatus or left uninfected and BALF or lungs were harvested for ex vivo stimulation or quantification of Adipoq or AdipoR expression by qRT[-PCR or flow cytometry. A. Expression of Adipoq and Adipor genes in lung homogenates and BALF cells from non-infected (NI) and conidia-infected WT mice. Lung and BALF were collected from the mice at 3 dpi. B. Summary of median fluorescence intensities of AdipoR1 staining on AMs, eosinophils, CD11c+SiglecF−, and CD11c−SiglecF− cells from non-infected (NI) and conidia-infected WT mice. C. qRT-PCR analysis for mRNA expression of Adipor1 and Adipor2 in lung homogenates. Wild-type (C57BL/6), Adipoq−/−, AdipoR1−/−, and AdipoR2−/−mice were neutrophil depleted and involuntarily aspirated A. fumigatus conidia. D. Flow cytometry staining of AdipoR1. Frequency of AdipoR1+ in ex-vivo cultured AMs in WT and Adipoq−/− mice. The histogram represents the AdipoR1 peak relative to IgG control in infected vs uninfected. E. qRT-PCR analysis for mRNA expression of Adipor1 and Adipor2 from the ex-vivo cultured AMs. The AMs were challenged with AF293 conidia with or without AdipoRon treatment. Data are a summary of two independently performed experiments. *p < 0.05, **p < 0.01, ***p < 0.001. (TIF) [file ppat.1012363.s002.tif]

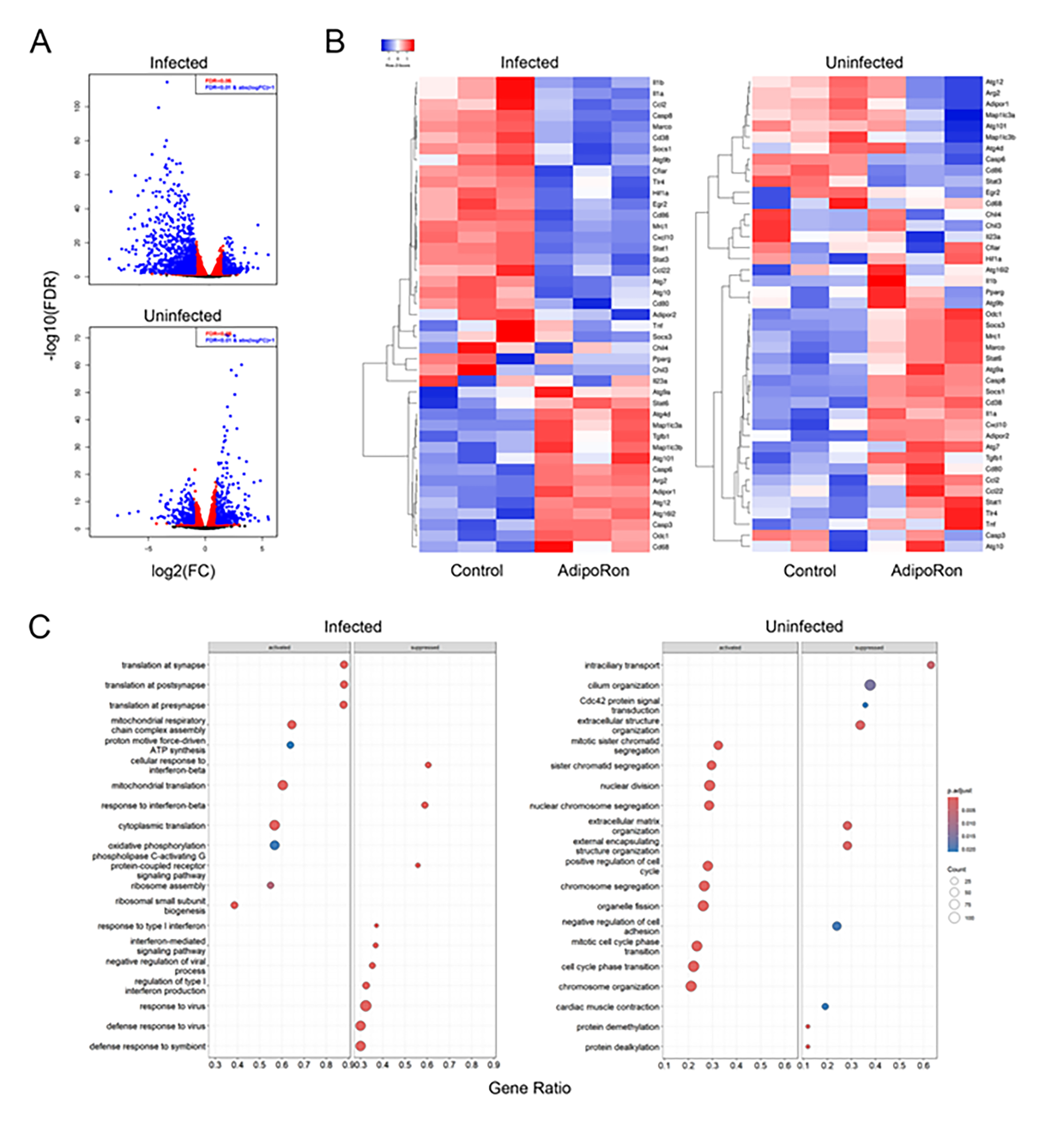

Supplement: S3 Fig — AMs were infected with swollen AF293 conidia with 1:9 cells/conidia for 10 hours, or left uninfected, with or without AdipoRon treatment, followed by RNA extraction for RNAseq analysis. A. Volcano plot depicting relative changes in gene expression of AdipoRon-treatment in APN-deficient AMs, infected (top) or uninfected (bottom). B. Heat map representation of the genes with highest differential expression in infected (left) and uninfected (right) APN-deficient AMs. C. GSEA-GO analysis of gene pathways that are both differentially expressed by AdipoRon treatment in infected (left) and uninfected (right) APN-deficient AMs. (TIF) [file ppat.1012363.s003.tif]

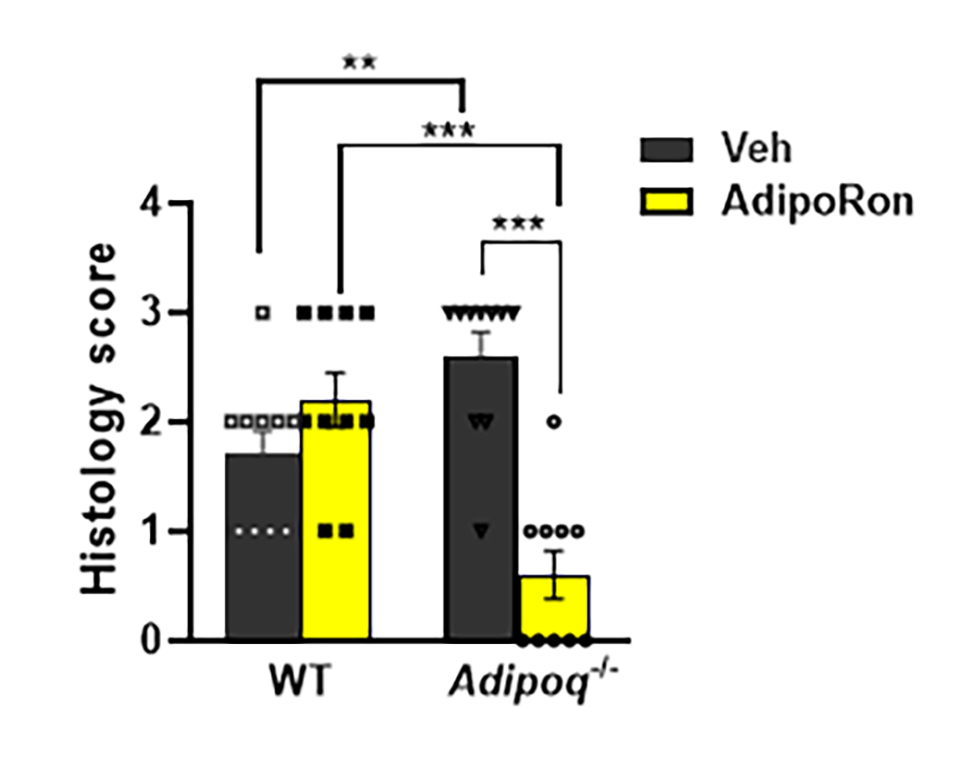

Supplement: S4 Fig — . Lung tissues were harvested 3 days post-infection with conidia from vehicle or AdipoRon-treated mice. Histological sections of lungs were stained with H&E. Results were summarized as the average of the combined scores for the distinct parameters evaluated. (TIF) [file ppat.1012363.s004.tif]

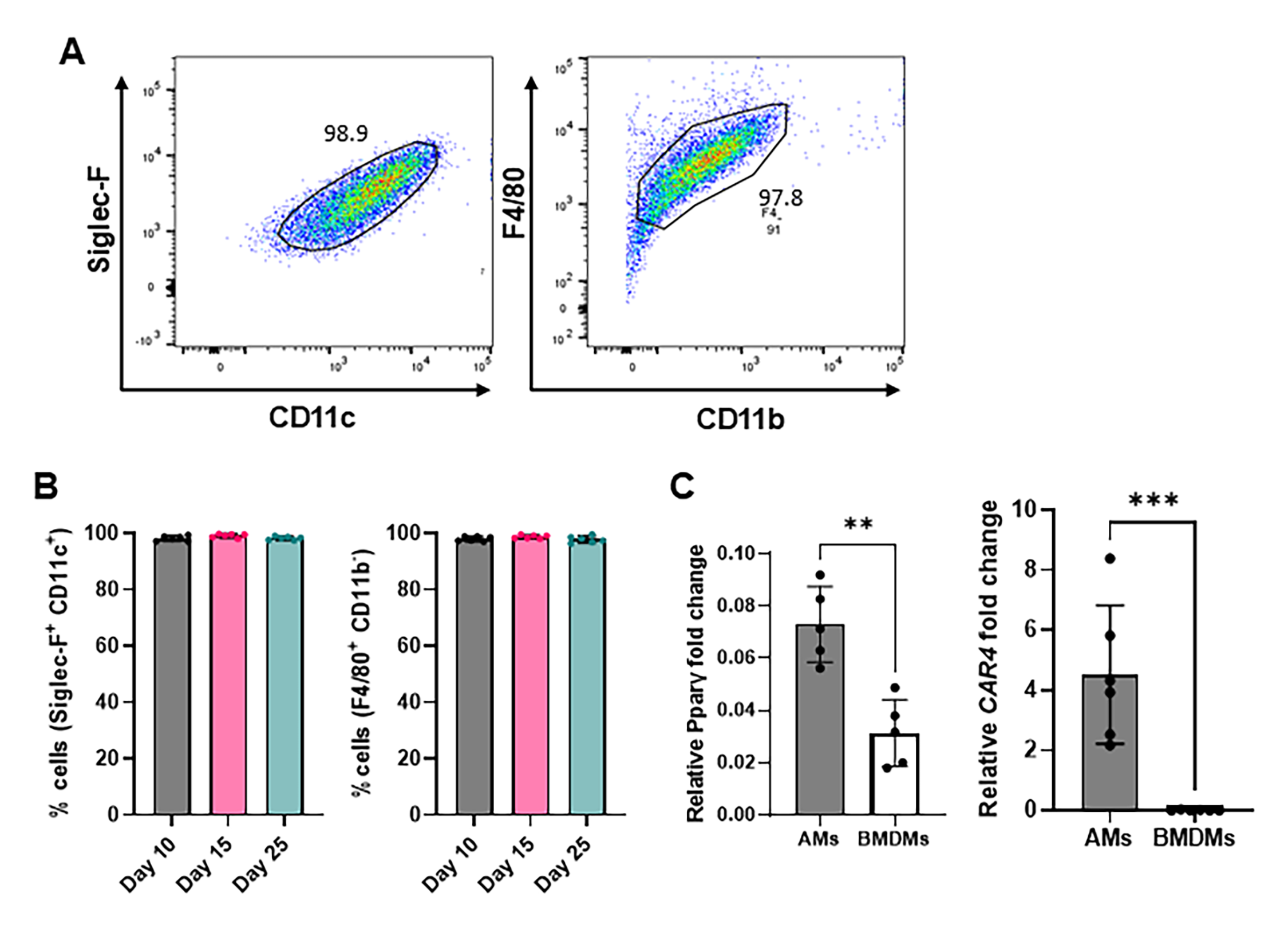

Supplement: S5 Fig — . AMs were grown as described in Materials and Methods and stained for Siglec-F, CD11c, F4/80 and CD11b or tested by qRT-PCR for expression of AM-associated genes. A. Representative flow cytometric dot plots. B. % cells Siglec-F+CD11c+ and F4/80+CD11b- was calculated from the data in S5A Fig. C. On day 15 of alveolar macrophage culture, the RNA was extracted followed by qRT-PCR analysis for the AMs markers: Pparγ and Car4. The relative fold change was calculated. (TIF) [file ppat.1012363.s005.tif]

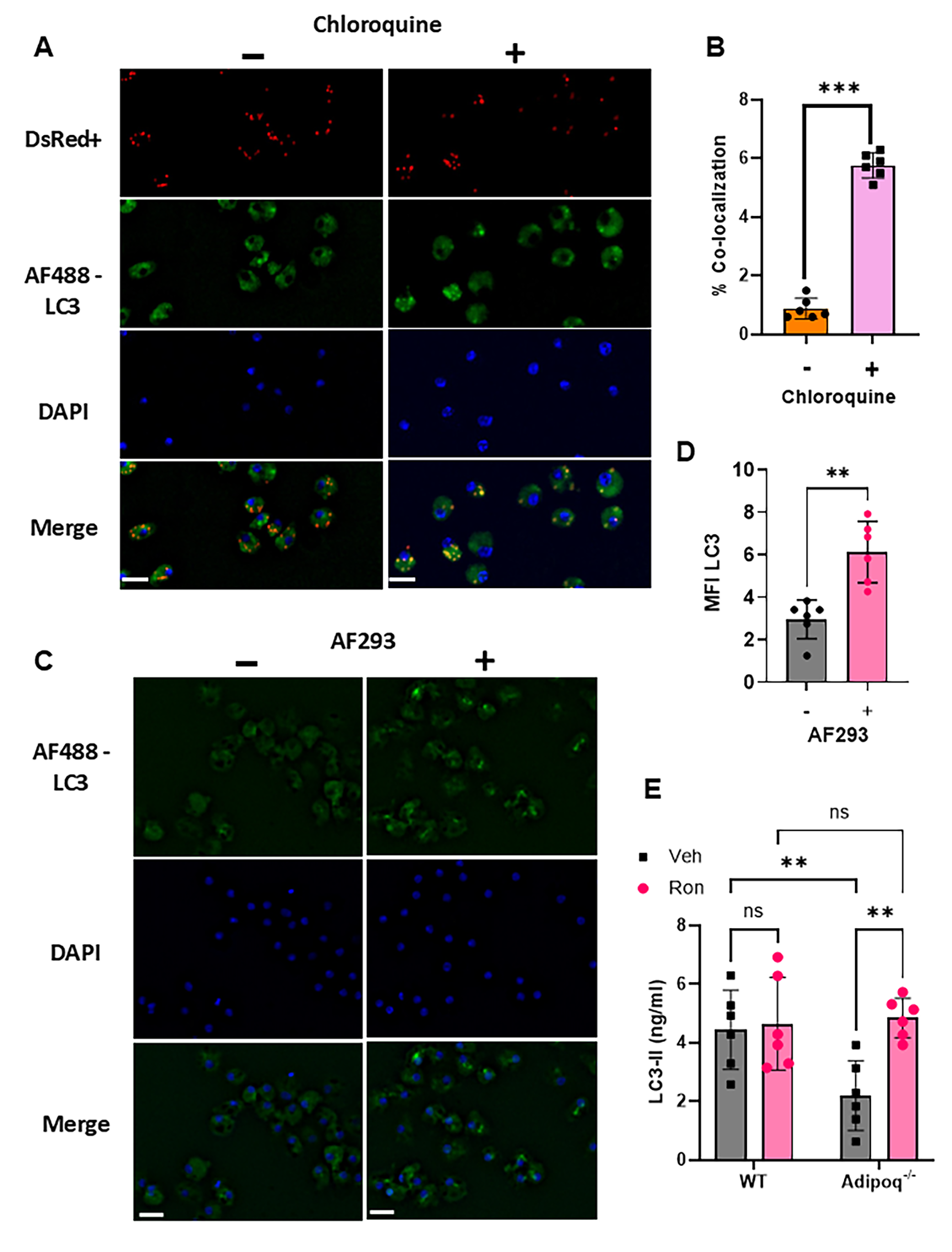

Supplement: S6 Fig — . AMs were cultured in ex vivo from WT, without and with Chloroquine, infection, or AdipoRon treatment. A. Microscopy of LAP-dsRed conidia and LC3+ AMs at a magnification of 20X. The images from DsRed, FITC, DAPI channels are presented followed by the merged image. Scale bar: 35µm. B. Microscopic quantification of % co-localization of LC3+ phagosome and DsRed+ conidia in ex vivo cultured AMs from WT infected group, without and with chloroquine treatment. C. Microscopy of LAP-AF293 conidia and LC3+ AMs at a magnification of 20X in ex vivo cultured AMs from WT uninfected group and WT infected group. The images from FITC, DAPI channels are presented followed by the merged image. Scale bar: 35µm. D. Mean Fluorescent Intensity of LC3 obtained from S6C Fig is graphed indicating the expression of LC3 without and with AF293 infection. E. LC3-II is quantified at the protein level using the ex-vivo cultured AMs by ELISA from cell lysates. Data are a summary of two independently performed experiments. *p < 0.05, **p < 0.01.d (TIF) [file ppat.1012363.s006.tif]
