# Supplementary material for: Adiponectin pathway activation dampens inflammation and enhances alveolar macrophage fungal killing via LC3-associated phagocytosis
Source: PLoS Pathog. 2025 Mar 17;21(3):e1012363. doi: 10.1371/journal.ppat.1012363 (PMC11949351; doi:10.1371/journal.ppat.1012363)
Supplement: S1 Table — Resources and reagents used in this study. (DOCX) [file ppat.1012363.s008.docx]

**S8 Table. Key Resources**

| **REAGENT or RESOURCE** | **SOURCE** | **IDENTIFIER** |
| --- | --- | --- |
| **Antibodies** | | |
| CD45-PerCP | BD Biosciences | 557235 |
| SiglecF-Superbright600 | Thermo Fisher Scientific | 63-1702-82 |
| SiglecF-PE | BD Biosciences | 552126 |
| Cd11c-SparekBlue550 | BioLegend | 117366 |
| Cd11c-PECy7 | Thermo Fisher Scientific | 25-0114-81 |
| Ly6G-BV785 | BioLegend | 127645 |
| Ly6G-FITC | BD Biosciences | 551460 |
| Fc Block | BD Biosciences | 553142 |
| Rabbit IgG - Isotype control | Abcam | ab171870 |
| Anti-AdipoR1 antibody | Abcam | ab70362 |
| Goat anti-Rabbit IgG H&L (Alexa Fluor 405) | Abcam | ab175652 |
| Anti-mouse EGR2 | Invitrogen | 247632 |
| Anti-mouse CD38 | Invitrogen | 2504972 |
| Goat anti-Rabbit IgG (H+L) Cross-Adsorbed Secondary Antibody, Alexa Fluor 488 | Thermo Fisher Scientific | A-11008 |
| Adiponectin receptor 1 Recombinant Rabbit Monoclonal Antibody | Invitrogen | MA5-32249 |
| **Chemicals** | | |
| Streptavidin, Alexa Fluor 633 conjugate | Invitrogen | S21375 |
| Biotin-XX, SSE | Invitrogen | B6352, B1606 |
| ACK lysing buffer | Thermo Fisher Scientific | A1049201 |
| Hygromycin B, *Streptomyces* sp. | Calbiochem | 31282-04-9 |
| Xylene | Millipore Sigma | XX0060 |
| TRIzol reagent | Thermo Fisher Scientific | 15596026 |
| AdipoRon | Cayman chemical | 924416-43-3 |
| Albumin, Bovine Serum | Millipore Sigma | 126579 |
| Normal Donkey Serum | abcam | ab7475 |
| Sodium Bicarbonate 7.5% solution | Thermo Fisher Scientific | 25080094 |
| Carboxymethylcellulose sodium salt | Thermo Fisher Scientific | A18105.36 |
| 0.5 M EDTA | Thermo Fisher Scientific | 15575020 |
| Dimethyl Sulfoxide | Millipore Sigma | D4540 |
| 10% Neutral Buffered Formalin | Thermo Fisher Scientific | 5701 |
| RPMI 1640, 1X with L-glutamine | CORNING | 28121001 |
| 2-Mercaptoehanol | A ALDRICH | SHBF0971V |
| 50ug Murine GM-CSF | PeproTech | 032255 K1622 |
| Lipofectamine 2000 Transfection Reagent | Thermo Fisher Scientific | 11668019 |
| Vectashield mounting medium for fluorescence with DAPI | VECTOR laboratories | H-1200-10 |
| FluoSpheres Polystyrene Microspheres, 1.0 μm, red fluorescent (580/605), for tracer studies | Thermo Fisher Scientific | F13083 |
| **Critical commercial assays** | | |
| Silver stain (Modified GMS) | Millipore Sigma | HT100A |
| RNeasy Mini kit | Qiagen | 74106 |
| RNase-Free DNase set | Qiagen | 79256 |
| PowerUp SYBR Green Master Mix | Thermo Fisher Scientific | A25742 |
| ABsolute Blue qPCR low ROX mix | Thermo Fisher Scientific | AB-4318 |
| High Capacity cDNA Reverse Transcription Kit | Thermo Fisher Scientific | 4368814 |
| Mouse Adiponectin/Acrp30 ELISA kit | R&D Systems | DY1119 |
| Murine TNF-α Standard TMB ELISA kit | Peprotech | 900-T54 |
| LC3B Antibody Kit for Autophagy | Invitrogen | L10382 |
| TriFECTa RNAi Kit | Integrated DNA Technologies | N/A |
| **Experimental models: Organism/strains** | | |
| *A. fumigatus strain* AF293 | Fungal Genetic Stock Center | Cat#A1100 |
| *A. fumigatus strain* AF293 ds-Red (FLARE strain) | Dr. Tobias Hohl, Memorial Sloan-Kettering | N/A |
| **Oligonucleotides** | | |
| 18S rRNA-encoding DNA primers and probe | 50-/56-FAM/AGC CAG CGG/ZEN/CCC GCAAAT G/3IABkFQ/-30 |  |
| **Software and algorithms** | | |
| FlowJo | FlowJo 10 | N/A |
| Prism 9 | Prism 9 | N/A |
| Gen5 | BioTek | N/A |
| Image J | Image J | N/A |
