# Supplementary material for: Adiponectin pathway activation dampens inflammation and enhances alveolar macrophage fungal killing via LC3-associated phagocytosis
Source: PLoS Pathog. 2025 Mar 17;21(3):e1012363. doi: 10.1371/journal.ppat.1012363 (PMC11949351; doi:10.1371/journal.ppat.1012363)
Supplement: S1 Data — All data tables have been exported into the supporting pdf file, with tables appearing in the order they are presented. (PDF) [file ppat.1012363.s009.pdf]

	X	Group A	Group B	Group C	Group D
	Days	WT (n=16)	*APN*$^{-/-}$ (n=15)	*AdipoR1*$^{-/-}$ (n=9)	*AdipoR2*$^{-/-}$ (n=8)
	X	Y	Y	Y	Y
1	3				1
2	4				1
3					
4	5				
5	5				
6	5				
7					
8	3				
9	3				
10	4				
11	6				
12					
13	2		1		
14	3		1		
15	3		1		
16	3		1		
17	3		1		
18	3		1		
19	4		1		
20					
21	4	1			
22	3	1			
23	5	1			
24	3	1			
25	6	0			
26	6	0			
27	6	0			
28	2	1			
29	2	1			
30	4	1			
31					
32	8	0			
33	2	1			
34	3	1			
35	4	1			
36	8	0			
37	8	0			
38	2			1	
39	2			1	
40	2			1	
41	2			1	
42	2			1	

	X	Group A	Group B	Group C	Group D
	Days	**WT (n=16)**	***APN*$^{-/-}$ (n=15)**	***AdipoR1*$^{-/-}$ (n=9)**	***AdipoR2*$^{-/-}$ (n=8)**
	X	Y	Y	Y	Y
43	3			1	
44	2				1
45	3				1
46	3				1
47	4				1
48	3				1
49	3		1		
50	3		1		
51	2		1		
52	3		1		
53	3		1		
54					
55	4		1		
56					
57	5		1		
58	6		1		
59	2			1	
60	2			1	
61	2			1	
62	3				1

	Group A	Group B	Group C	Group D
	**WT**	***APN*$^{-/-}$**	***AdipoR1*$^{-/-}$**	***AdipoR2*$^{-/-}$**
1	1572	1879	46081	1570
2	476	1163	16192	574
3	247	3910	401179	95752
4	279	2867	176768	114
5		2067	87037	627
6	729	1966		117
7	1576		94673	

	Group A	Group B	Group C	Group D
	**WT**	***APN***$^{-/-}$	***AdipoR1***$^{-/-}$	***AdipoR2***$^{-/-}$
1	0.7	23.2	46.0	11.4
2	3.4	22.1	25.1	9.3
3	3.7	25.6	18.4	13.8
4	1.4	16.7	11.5	3.5
5	4.5	25.8	9.0	9.1
6	0.9	22.3	16.9	1.8
7	1.1	11.9	17.4	5.1
8	5.2	18.7	23.0	3.8
9	4.0	21.8	27.4	14.7
10	3.1	18.2	9.8	5.6

	Group A	Group B	Group C	Group D
	WT	*Adipoq*$^{-/-}$	*AdipoR1*$^{-/-}$	*AdipoR2*$^{-/-}$
1				
2				
3				
4				
5				
6	911049	4935000	4535553	3607135
7	1798291	7696192	3810021	2513073
8	1356979	5681910	1768998	2403968
9	774434	4742181	4478653	2109783
10	890174	3081682	5311872	3180283
11	1951824	1090621	3923691	2998105

	Group A	Group B	Group C	Group D
	WT	*Adipoq*$^{-/-}$	*AdipoR1*$^{-/-}$	*AdipoR2*$^{-/-}$
1				
2				
3				
4				
5	96593	1327120	789753	1101626
6	109283	1213566	273923	667236
7	573259	132974	152518	349383
8	278269	1129373	416215	529743
9	164016	629113	391837	815383
10	114982	1037924	102735	410389

	Group A	Group B	Group C	Group D
	**WT**	***Adipoq*** $^{-/-}$	***AdipoR1*** $^{-/-}$	***AdipoR2*** $^{-/-}$
1				
2				
3				
4				
5				
6	22400	178967		
7	31801	433197	224741	319273
8	211932	523915	72918	446318
9	174399	619379	46281	360405
10	115254	207058	123438	159347
11	191739	99932	112937	292730
12			152983	610837

	Group A	Group B	Group C	Group D	Group E
	**WT**	***Adipoq*** $^{-/-}$	***AdipoR1*** $^{-/-}$	***AdipoR2*** $^{-/-}$	Title
1	40719	879832	780727	417980	
2	200182	951970	383691	311702	
3	199772	1619273	184073	318694	
4	197481	219273	677351	529743	
5	110821	485578	592739	129739	
6	51973	719281	319374	429710	

	Group A	Group B	Group C	Group D	Group E
	**WT**	***Adipoq***$^{-/-}$	***AdipoR1***$^{-/-}$	***AdipoR2***$^{-/-}$	Title
1	672821	2324918	2721264	1576282	
2	219374	4777457	5923803	1095826	
3	750448	3792013	1370906	1534453	
4	630914	1927374	3239701	1937364	
5	348718	1731588	2137993	2018363	
6	810383	816391	1138793	1130283	

	Group A	Group B	Group C	Group D	Group E	Group F
	**WT**	***Adipoq*** $^{-/-}$	***AdipoR1*** $^{-/-}$	***AdipoR2*** $^{-/-}$	Title	Title
1		4.915601	2.160978	1.895419		
2		2.220454	3.493032			
3	0.937071	4.897445	11.892496	1.066926		
4	0.376567		5.468126	0.886438		
5	1.073859		2.120901	3.211000		
6	0.759134	1.064973	5.670983	12.298000		
7	1.024000	1.416073	0.150463	0.737000		
8						
9	0.611000	3.783000				
10						
11		4.627000				
12	1.287000					

	Group A	Group B	Group C	Group D	Group E	Group F
	**WT**	***Adipoq*** $^{-/-}$	***AdipoR1*** $^{-/-}$	***AdipoR2*** $^{-/-}$	Title	Title
1	1.297844			1.960000		
2	2.128725			6.729000		
3	0.703386			0.456000		
4	0.700927					
5	1.768002					
6	0.415249					
7	0.628000					
8						
9	0.599500	3.779221	3.738856	7.886931		
10		9.344833	4.704017	1.472057		
11	1.105500	24.884934	54.346065	2.344151		
12		16.060123	81.857428	1.764828		
13		2.441123	55.259263			
14		2.385674	50.911976			
15		2.417139				
16						
17		2.361500				
18						
19		0.797000				

	Group A	Group B	Group C	Group D	Group E	Group F
	**WT**	***Adipoq*** $^{-/-}$	***AdipoR1*** $^{-/-}$	***AdipoR2*** $^{-/-}$	Title	Title
1	0.264000	1.291500		2.798000		
2				2.173000		
3	0.739000	0.367000		0.251000		
4						
5	0.597000		79.236293			
6						
7			1.882872			
8						
9	1.962590	1.950686	4.422860	2.744325		
10	0.487135	2.416995		0.993194		
11	0.819147		7.384090	0.907916		
12		3.123866	15.328966	1.264784		
13	1.729635	2.331922				
14	1.432317	3.496780	6.085654			
15	0.515425	4.234569				

	Group A	Group B	Group C	Group D	Group E	Group F
	**WT**	***Adipoq*** $^{-/-}$	***AdipoR1*** $^{-/-}$	***AdipoR2*** $^{-/-}$	Title	Title
1	1.468000	1.719500		0.736000		
2				0.874000		
3	0.660500	1.242000		1.602000		
4						
5	1.501000					
6						
7						
8						
9	1.268388	3.317237	0.519350	1.195783		
10	1.712153	1.257226	1.295507	0.254869		
11	2.026048	2.532086	3.201817	1.090031		
12	0.307386	0.257239	4.207240	0.337642		
13	1.146521	0.689487	2.851521			
14	0.644896	0.549204	1.180896			
15		0.733627				

	Group A	Group B	Group C	Group D	Group E	Group F
	**WT**	***Adipoq*** $^{-/-}$	***AdipoR1*** $^{-/-}$	***AdipoR2*** $^{-/-}$	Title	Title
1	0.457000	1.250000		6.370000		
2	0.470000	1.577000		*13.120000**		
3	1.348000			0.495000		
4	0.489000	1.581000		27.528507		
5	1.380000					
6						
7	2.351000					
8						
9	0.913787	5.077537	10.675808			
10		28.320033		10.319134		
11	2.821161	110.582255	280.232202	5.152013		
12	0.021212	14.085432	206.021387	2.048865		
13		6.666983	271.472059			
14	2.803698	2.856929	40.106396			
15		2.852206	50.743000			

	Group A	Group B	Group C	Group D	Group E	Group F
	**WT**	***Adipoq*** $^{-/-}$	***AdipoR1*** $^{-/-}$	***AdipoR2*** $^{-/-}$	Title	Title
1	0.463500	1.413500		6.370000		
2						
3	0.918500	1.281000		0.495000		
4						
5	1.865500					
6						
7						
8						
9	1.396570	3.191470	1.985347	1.917220		
10	1.125677	2.028949	2.169034	0.647953		
11	1.149269	4.106804	4.406028	1.083342		
12	0.831232	1.784184	7.394866	0.870920		
13	1.171733	1.414907	3.500630			
14	0.568265	1.220121	3.702396			
15		1.184022				

	Group A	Group B	Group C	Group D	Group E	Group F
	**WT**	***Adipoq*** $^{-/-}$	***AdipoR1*** $^{-/-}$	***AdipoR2*** $^{-/-}$	Title	Title
1		994.000000	4913.000000	881.0000000		
2	994.000000	13748.00000		212.0000000		
3		1260.000000	25740.00000	740.0000000		
4	1872.000000	6997.000000	22108.00000	199.0000000		
5		10373.00000	3713.000000	16564.00000		
6		2813.000000		466.0000000		
7	1631.000000	342.000000	9260.000000	11096.00000		
8			8710.000000	4543.000000		
9				69.9109792		
10	1615.000000			456.2611280		
11				353.2344210		
12	437.000000					
13	54.000000					
14						
15	1863.000000					
16	6421.000000					
17						
18						
19						
20	353.234421	198.694362	5401.543030			
21	224.451039					
22			4809.139470			
23						
24						
25						
26						
27						
28	662.314540					
29						
30		4989.436200				
31		636.557864				

Table format: **Grouped**		Group A NI							
		A:1	A:2	A:3	A:4	A:5	A:6	B:1	B:2
1	**WT**	1.000000	1.000000	1.00000	1.0000	1.00000	1.00000	1.450	2.21000
2	***Adipoq***$^{-/-}$	35.382240	39.383040	32.38300	36.2847	35.29000	37.13500	500.373	505.28434
3	***AdipoR1***$^{-/-}$	2.192790	1.237900	4.28823	3.2730	1.92746	1.13874	281.287	219.28710
4	***AdipoR2***$^{-/-}$	28.443000	29.474000	25.49840	25.3957	30.29746	21.39470	222.861	215.38300

	Group B			
	Infected			
	B:3	B:4	B:5	B:6
1	1.890	1.93794	2.18370	1.1847
2	501.383	520.29470	489.93740	510.2973
3	214.387	250.29470	267.39730	220.1928
4	220.383	211.29370	242.93824	201.2937

Table format: **Grouped**		Group A							
		NI							
		A:1	A:2	A:3	A:4	A:5	A:6	B:1	B:2
1	**WT**	1.000000	1.0000000	1.0000000	1.000000	1.00000	1.0000000	7.8900	5.2100
2	***Adipoq$^{-/-}$***	7.340484	8.3704000	10.3834000	12.197300	5.93791	6.1383000	204.3739	210.3830
3	***AdipoR1$^{-/-}$***	5.746200	8.4462000	4.4892000	10.237000	4.68300	15.3745283	230.4720	240.2974
4	***AdipoR2$^{-/-}$***	4.112455	8.3740000	3.3984840	10.329746	4.29472	6.9372084	155.8770	158.4743

	Group B			
	infected			
	B:3	B:4	B:5	B:6
1	2.34000	6.3850	8.39475	3.2937465
2	209.43874	196.3957	221.39470	211.3847000
3	234.46828	241.3470	216.38400	200.2947000
4	155.37300	156.3850	169.39750	132.3987500

Table format: **Grouped**		Group A							
		NI							
		A:1	A:2	A:3	A:4	A:5	A:6	B:1	B:2
1	**WT**	1.000000	1.0000000	1.0000000	1.000000	1.00000	1.0000000	7.8900	5.2100
2	***Adipoq*** −/−	7.340484	8.3704000	10.3834000	12.197300	5.93791	6.1383000	204.3739	210.3830
3	***AdipoR1*** −/−	5.746200	8.4462000	4.4892000	10.237000	4.68300	15.3745283	230.4720	240.2974
4	***AdipoR2*** −/−	4.112455	8.3740000	3.3984840	10.329746	4.29472	6.9372084	155.8770	158.4743

	Group B			
	infected			
	B:3	B:4	B:5	B:6
1	2.34000	6.3850	8.39475	3.2937465
2	209.43874	196.3957	221.39470	211.3847000
3	234.46828	241.3470	216.38400	200.2947000
4	155.37300	156.3850	169.39750	132.3987500

Table format: **Grouped**		Group A							
		NI							
		A:1	A:2	A:3	A:4	A:5	A:6	B:1	B:2
1	**WT**	1.000000	1.000000	1.000000	1.000000	1.000000	1.000000	101.125000	105.383000
2	***Adipoq*** $^{-/-}$	500.340480	510.374600	496.474000	551.297300	498.294740	521.394600	100.380470	105.373030
3	***AdipoR1*** $^{-/-}$	150.237000	148.392700	143.294700	156.274000	141.286000	159.397400	129.386000	131.381000
4	***AdipoR2*** $^{-/-}$	243.496000	240.474700	239.487400	250.297400	231.864000	278.919740	268.727000	261.837000

	Group B			
	Infected			
	B:3	B:4	B:5	B:6
1	120.373900	110.309740	132.108400	121.297400
2	102.438300	131.294000	105.294000	111.724170
3	128.381000	120.397400	139.297400	116.298500
4	260.383000	271.297000	275.297500	281.397300

Table format: **Grouped**		Group A							
		NI							
		A:1	A:2	A:3	A:4	A:5	A:6	B:1	B:2
1	**WT**	1.000000	1.000000	1.000000	1.000000	1.000000	1.000000	2.100000	1.100000
2	***Adipoq***$^{-/-}$	274.487400	273.495700	278.484000	280.540000	250.287000	472.397000	1843.394000	1849.383000
3	***AdipoR1***$^{-/-}$	281.193700	251.281000	259.202800	340.970000	450.398400	523.274000	1382.293700	1298.389170
4	***AdipoR2***$^{-/-}$	263.197000	261.387400	267.596000	482.984000	692.274000	350.274000	1663.493000	1663.393700

	Group B			
	Infected			
	B:3	B:4	B:5	B:6
1	2.450000	6.394700	1.588700	1.549000
2	1830.386000	1587.759000	1675.865000	1843.754000
3	1182.018200	1532.754000	980.847000	1965.865400
4	1659.398300	1432.557000	985.589900	1854.754000

Table format: **Grouped**		Group A NI							
		A:1	A:2	A:3	A:4	A:5	A:6	B:1	B:2
1	**WT**	1.000000	1.000000	1.000000	1.000000	1.000000	1.000000	13.269000	12.384000
2	***Adipoq*** $^{-/-}$	30.383000	28.474000	34.484700	176.865000	210.968900	121.865000	1327.380300	1329.383000
3	***AdipoR1*** $^{-/-}$	5.287490	4.289740	8.283800	9.135700	3.158600	10.749000	1092.283700	1021.625710
4	***AdipoR2*** $^{-/-}$	41.642940	45.584000	40.339000	67.865000	21.646900	48.858000	1192.687000	1199.383700

	Group B			
	Infected			
	B:3	B:4	B:5	B:6
1	15.387360	5.744000	2.136000	8.176000
2	1340.383000	1754.432000	1213.644300	1100.754000
3	1187.273600	1324.750000	1098.754000	1436.750000
4	1189.383000	1326.646000	1254.754300	1021.754000

Table format: **Grouped**		Group A							
		NI							
		A:1	A:2	A:3	A:4	A:5	A:6	B:1	B:2
1	**WT**	1.000000	1.000000	1.000000	1.000000	1.000000	1.000000	17.270000	20.376430
2	***Adipoq*** $^{-/-}$	8.348404	7.448000	9.474030	10.740000	5.742200	6.532000	649.870800	650.393700
3	***AdipoR1*** $^{-/-}$	9.862000	11.372000	7.203000	10.854000	15.643000	21.743000	875.297000	763.297000
4	***AdipoR2*** $^{-/-}$	2.949000	5.473700	3.474000	8.853000	4.426000	2.632000	552.600000	559.383900

	Group B			
	Infected			
	B:3	B:4	B:5	B:6
1	18.379032	25.283000	19.273000	32.186300
2	658.380300	732.186300	425.297300	693.283000
3	812.376000	657.287400	710.823000	821.973400
4	554.383700	450.283000	599.183400	421.729000

Table format: **Grouped**		Group A							
		NI							
		A:1	A:2	A:3	A:4	A:5	A:6	B:1	B:2
1	**WT**	50.15	79.740000	49.84	72.37	45.93	56.71	156.28	124.25
2	***Adipoq*** $^{-/-}$	52.48	71.190000	54.98	79.74	61.67	68.32	114.77	167.71
3	***AdipoR1*** $^{-/-}$	60.13	61.430000	61.28	62.06	59.91	65.45	148.23	102.85
4	***AdipoR2*** $^{-/-}$	58.19	52.750000	55.75	53.62	59.12	55.88	126.62	132.33

	Group B			
	Infected			
	B:3	B:4	B:5	B:6
1	144.36	126.31	150.34	138.77
2	135.37	195.82	178.43	123.77
3	156.09	133.84	110.65	140.21
4	128.39	140.76	144.21	120.49

Table format: **Grouped**		Group A							
		NI							
		A:1	A:2	A:3	A:4	A:5	A:6	B:1	B:2
1	**WT**	9.397	3.7394	17.3297	4.92	21.210	7.900	48.240	31.3730
2	***Adipoq***$^{-/-}$	13.264	20.2746	7.3794	10.38	15.340	15.230	63.374	74.3874
3	***AdipoR1***$^{-/-}$	3.374	9.3840	19.7300	15.39	18.320	12.680	44.370	53.2700
4	***AdipoR2***$^{-/-}$	18.340	6.3750	8.3730	10.38	5.245	15.210	56.375	46.2824

	Group B			
	Infected			
	B:3	B:4	B:5	B:6
1	51.37300	40.297	55.283	39.780
2	59.19000	60.297	75.240	66.210
3	63.47900	51.470	49.560	54.230
4	60.38648	54.280	47.840	61.110

Table format: **Grouped**		Group A							
		NI							
		A:1	A:2	A:3	A:4	A:5	A:6	B:1	B:2
1	**WT**	10.3692	6.2739	19.3830	7.3974	21.8700	7.1290	5.3975	17.3759
2	***Adipoq*** $^{-/-}$	15.2490	5.2974	12.3840	7.2300	10.3240	12.7760	62.3800	71.3790
3	***AdipoR1*** $^{-/-}$	16.2736	28.3746	6.9370	10.3847	30.1930	9.1100	61.3400	42.8400
4	***AdipoR2*** $^{-/-}$	8.3819	5.2940	17.3900	12.3804	16.3300	23.1380	10.3930	29.3794

	Group B			
	Infected			
	B:3	B:4	B:5	B:6
1	46.38490	39.3847	26.2480	22.1937
2	46.00000	50.3895	67.2874	48.2890
3	50.28400	47.3900	55.2940	60.1730
4	38.20180	32.7230	41.2970	18.2260

Table format: **Grouped**		Group A							
		WT							
		A:1	A:2	A:3	A:4	A:5	A:6	B:1	B:2
1	Control	22.9	22.60	22.70	23.67	23.19	23.44	34.9	34.1

	Group B			
	Adipoq$^{-/-}$			
	B:3	B:4	B:5	B:6
1	34.7	35.68	35.13	35.22

Table format: **Grouped**		Group A							
		WT							
		A:1	A:2	A:3	A:4	A:5	A:6	B:1	B:2
1	Control	5.19	5.10	5.94	5.60	5.73	5.46	4.38	4.31

	Group B *Adipoq*$^{-/-}$			
	B:3	B:4	B:5	B:6
1	4.28	4.20	4.11	3.80

Table format: **Grouped**		Group A							
		WT							
		A:1	A:2	A:3	A:4	A:5	A:6	B:1	B:2
1	Control	34.8	34.1	35.1	33.65	34.19	33.7	33.5	33.1

	Group B *Adipoq*$^{-/-}$			
	B:3	B:4	B:5	B:6
1	33.6	32.91	33.76	34.1

Table format: **Grouped**		Group A							
		WT							
		A:1	A:2	A:3	A:4	A:5	A:6	B:1	B:2
1	Control	19.9	21.4	22.4	22.1	21.9	22.5	27.4	27.1

	Group B			
	Adipoq$^{-/-}$			
	B:3	B:4	B:5	B:6
1	27.9	27.8	27.0	27.7

Table format: **Grouped**		Group A							
		WT							
		A:1	A:2	A:3	A:4	A:5	A:6	B:1	B:2
1	Control	0.84	1.00	0.91	0.79	1.10	0.69	0.94	1.00

	Group B			
	Adipoq$^{-/-}$			
	B:3	B:4	B:5	B:6
1	1.48	1.43	1.21	0.78

Table format: **Grouped**		Group A							
		WT							
		A:1	A:2	A:3	A:4	A:5	A:6	B:1	B:2
1	Control	16.90	16.3	17.10	16.58	17.21	16.80	21.70	21.9

	Group B			
	Adipoq$^{-/-}$			
	B:3	B:4	B:5	B:6
1	21.60	22.10	21.50	21.30

	Group A	Group B
	WT	*Adipoq*$^{-/-}$
1	9.1019000	65.8462000
2	10.4732000	40.5278000
3	10.4732000	43.1894000
4	9.1397400	56.2750000
5	8.5297500	45.2826500
6	11.2467000	60.9570000

	Group A	Group B
	WT	*Adipoq*$^{-/-}$
1	75.3940000	89.7000000
2	80.1500000	87.0300000
3	82.9800000	89.0500000
4	71.5200000	92.1974000
5	88.1900000	90.1800000
6	78.2400000	84.2947000

	Group A	Group B
	WT	*Adipoq*$^{-/-}$
1	59.1200	54.210
2	61.2930	56.220
3	53.2900	48.282
4	70.2400	51.397
5	55.2740	45.294
6	65.2900	60.290

	Group A	Group B	Group C	Group D
	WT	*Adipoq*$^{-/-}$	WT	*Adipoq*$^{-/-}$
1		29	12	
2		27	7	
3	27	38	17	
4	35	42	17	
5				24
6				20
7	36	40		32
8	41	40		64
9	28	29		52
10	28			
11	36		28	38
12			18	14
13			8	

	Group A	Group B	Group C	Group D
	WT	*Adipoq*$^{-/-}$	WT	*Adipoq*$^{-/-}$
1		70.70	71.45	
2		78.30	79.09	
3	52.00	50.70	80.80	
4	63.00	70.40	74.30	
5				65.50
6	89.50	71.00		75.20
7	78.80	72.80		76.20
8	80.70	55.60		83.50
9	67.10			69.90
10	66.30			
11			79.40	
12				60.00
13			86.80	80.30
14			85.73	

	Group A	Group B	Group C	Group D
	**WT**	***Adipoq***$^{-/-}$	Title	Title
1		34.60		
2		31.60		
3	30.80	23.30		
4	42.40	25.30		
5	36.80	33.00		
6	45.50	38.40		

	X	Group A	Group B	Group C	Group D
	Days	/VT Veh (n=10)	WT Ron (n=9)	Title	Title
	X	Y	Y	Y	Y
1	2	1	1		
2	2				
3	2				
4	3	1	1		
5	3		1		
6					
7	4	1	1		
8	5				
9	6				
10	7	0	0		
11	7	0			
12	7	0			
13	2	1	1		
14	2		1		
15	2				
16	3	1	1		
17	3	1	1		
18	3	1			

	X	Group A	Group B	Group C	Group D
	Days	Title	Title	*poq*$^{-/-}$ Veh (n=	*poq*$^{-/-}$ Ron (n=
	X	Y	Y	Y	Y
1	2			1	1
2	2			1	
3	2			1	
4	3			1	1
5	3				1
6					
7	4				1
8	5			1	
9	6				1
10	7			0	0
11	7				0
12	7				
13	2			1	1
14	2			1	1
15	2			1	
16	3			1	1
17	3			1	
18	3				

	Group A	Group B	Group C	Group D	Group E
	Veh	AdipoRon		Veh	AdipoRon
1					
2					
3	72.100000	5.30000000		945.2000000	79.90000000
4	70.300000	1.50000000		*3748.000000*	130.7000000
5	*68346.00000*	13.30000000		1419.000000	19.80000000
6					
7	0.400000	6.00000000		*2.2100000**	0.30000000
8	24.900000	119.4000000(			13.50000000
9		0.80000000		48.5000000	2.90000000
10		125.8000000		35.1000000	5.40000000
11					
12	*'454.000000**	27.80000000		550.6000000	10.90000000
13	9.300000	218.5000000		37.9000000	41.70000000
14		*1100.000000*			

Table format: **Grouped**		Group A							
		Title							
		A:1	A:2	A:3	A:4	A:5	A:6	A:7	A:8
1	WT								
2	*Adipoq*$^{-/-}$								

	Group B								
	Veh								
	B:1	B:2	B:3	B:4	B:5	B:6	B:7	B:8	C:1
1	0.287	1.009	1.1950	1.439	1.6900	1.460	2.0170	2.71538	1.847
2	11.533	37.879	11.9810	15.826	16.1937	13.927	18.1837	12.39080	1.507

	Group C						
	Ron						
	C:2	C:3	C:4	C:5	C:6	C:7	C:8
1	3.504	5.757	4.485	1.518	5.463	0.1620	1.4910
2	3.377	1.275	3.126	3.138	2.654	3.9173	2.3819

	Group A	Group B	Group C	Group D	Group E
	**Veh**	**AdipoRon**		**Veh**	**AdipoRon**
1	211765.0000	189880.0000		158212.0000	210101.0000
2	274847.0000	248884.0000		303195.0000	268268.0000
3	273289.0000	169942.0000		265758.0000	248972.0000
4	229037.0000	239877.0000		119140.0000	281148.0000
5	219283.0000	230325.0000		258271.0000	251038.0000
6	261088.0000	214497.0000		280838.0000	221038.0000

	Group A	Group B	Group C	Group D	Group E
	**Veh**	**AdipoRon**		**Veh**	**AdipoRon**
1	29631.00000	15988.00000		32021.00000	18426.00000
2	53290.00000	44220.00000		87627.00000	20603.00000
3	31937.00000	8971.000000		21025.00000	21917.00000
4					
5	10397.00000	22345.00000		27620.00000	23167.00000
6	43319.00000	31313.00000		62363.00000	18705.00000
7	31830.00000	13597.00000		15939.00000	15298.00000

	Group A	Group B	Group C	Group D	Group E
	**Veh**	**AdipoRon**		**Veh**	**AdipoRon**
1	39552.00000	81161.00000		13583.00000	127510.0000
2	56792.00000	133737.0000		72065.00000	152201.0000
3	41972.00000	83642.00000		130791.0000	88312.00000
4		104752.0000		8187.000000	105956.0000
5	68864.00000	185837.0000		36441.00000	110833.0000
6	37508.00000	107424.0000		85896.00000	71359.00000
7	35183.00000				

	Group A	Group B	Group C	Group D	Group E
	**Veh**	**AdipoRon**		**Veh**	**AdipoRon**
1	70613.00000	126087.0000		17115.00000	235408.0000
2	99004.00000	72732.00000		111599.0000	149409.0000
3	139049.0000	127367.0000		144639.0000	207291.0000
4	78859.00000	115432.0000		29479.00000	120533.0000
5	92739.00000	91742.00000		105948.0000	162937.0000
6	102837.0000	114782.0000		114221.0000	197392.0000

	Group A	Group B	Group C	Group D	Group E
	**Veh**	**AdipoRon**		**Veh**	**AdipoRon**
1	284172.0000	122341.0000		142029.0000	240393.0000
2	605826.0000	420260.0000		448415.0000	210317.0000
3	253224.0000	104778.0000		261370.0000	314513.0000
4	454077.0000	185233.0000		247934.0000	227155.0000
5	192703.0000	360542.0000		622041.0000	301379.0000
6	592129.0000	159612.0000		231195.0000	231937.0000

	Group A	Group B	Group C	Group D	Group E
	Veh	AdipoRon		Veh	AdipoRon
1	0.960000	0.46000000		1.7700000	0.54000000
2	1.040000	0.44000000			0.29000000
3	0.960000	0.48000000		2.4300000	0.28000000
4	1.040000	0.16000000			0.27000000
5	1.030000	0.18000000		1.9300000	0.29000000
6	1.010000	0.14000000			
7	0.710000	0.15000000		1.9200000	0.53000000
8	1.190000	0.57000000			
9		0.78000000		0.8950000	0.52500000
10					
11				0.8950000	0.28500000
12					
13				2.8900000	
14				2.2800000	
15				1.0700000	0.33000000

	Group A	Group B	Group C	Group D	Group E	Group F
	**Veh**	**AdipoRon**	Title	**Veh**	**AdipoRon**	Title
1	0.950000	2.57000000		8.6000000	0.69000000	
2	1.060000	2.60000000		6.9800000	0.68000000	
3	0.950000	2.43000000		2.1900000	0.51000000	
4	1.060000	0.77000000		26.9500000	0.65000000	
5	1.005000	0.70000000		13.3200000	1.98000000	
6	1.005000	0.56000000		3.8400000	1.71000000	
7	1.720000	0.60000000		10.5350000	1.86000000	
8	0.340000	1.19000000		15.6600000	1.94000000	
9		1.13000000		7.8000000		

	Group A	Group B	Group C	Group D	Group E	Group F
	**Veh**	**AdipoRon**	Title	**Veh**	**AdipoRon**	Title
1	1.080000	0.32500000		0.5850000	0.78000000	
2	0.930000				0.58000000	
3	1.080000	0.28000000		0.7100000	0.68000000	
4	0.930000					
5	1.005000	0.13500000		0.6900000	0.61000000	
6						
7	1.005000	0.10000000		0.6450000	0.11000000	
8	0.950000					
9	1.120000	0.24500000		1.3650000		
10	1.210000				0.58000000	
11	0.940000	0.56500000		1.3950000	0.11000000	
12		0.26000000		0.6700000	0.55000000	
13		0.09000000		0.6100000		
14		0.54000000		1.3200000		

	Group A	Group B	Group C	Group D	Group E	Group F
	**Veh**	**AdipoRon**	Title	**Veh**	**AdipoRon**	Title
1	1.060000	9.02500000		6.4850000	2.07000000	
2	1.180000					
3	0.800000	8.82500000		6.3350000	1.42500000	
4	1.000000					
5	1.120000	9.24500000		4.6250000	8.00000000	
6	0.900000					
7		8.04500000		4.6450000	6.53500000	
8						
9		5.91000000		1.6150000		
10						
11		6.10500000		1.5750000		
12		9.03000000		6.4900000	2.07000000	
13		9.25000000		4.6300000	8.00000000	
14		5.91000000		*1.6100000**		

	Group A	Group B	Group C	Group D	Group E	Group F
	**Veh**	**AdipoRon**	Title	**Veh**	**AdipoRon**	Title
1	0.950000	4.03000000		37.0350000	0.92000000	
2	1.250000					
3	0.980000	3.98500000		38.2850000	0.71000000	
4	0.860000					
5	1.100000	1.70500000		34.5400000	2.64000000	
6	0.920000					
7		1.38500000		38.8500000	1.87000000	
8						
9		1.91500000		2.1850000		
10						
11		1.71000000		1.4800000		
12		4.03000000		36.9400000	0.98000000	
13		1.71000000		34.8300000	2.84000000	
14		1.91000000				

	Group A	Group B	Group C	Group D	Group E	Group F
	**Veh**	**AdipoRon**	Title	**Veh**	**AdipoRon**	Title
1	0.950000	0.96000000		1.3450000	0.83500000	
2	1.020000					
3	1.030000	0.98500000		1.5250000	0.45500000	
4	0.990000					
5		0.53500000		1.7500000	0.59500000	
6						
7		0.47500000		1.7300000	0.50500000	
8						
9		1.05000000		1.3400000		
10						
11		1.22000000		1.2250000		
12	0.990000	0.96000000		1.3500000	0.84000000	
13	1.010000	0.54000000		1.7500000	0.60000000	
14		1.05000000				

	Group A	Group B	Group C	Group D	Group E
	**Veh**	**AdipoRon**	Title	**Veh**	**AdipoRon**
1	4631.000000	3656.000000		3662.000000	2628.000000
2	3427.000000	3394.000000		2195.000000	592.0000000
3	8540.000000	4568.000000		6002.000000	3595.000000
4	10408.00000	5847.000000		5458.000000	5989.000000
5	9922.000000	915.0000000		9894.000000	587.0000000
6	6741.029000	5743.000000		10152.00000	3654.000000
7				11055.00000[illegible]	112.0000000[illegible]

Table format: **Grouped**		Group A WT							
		A:1	A:2	A:3	A:4	A:5	A:6	B:1	B:2
1	**WT**	15.2974000	29.2974000	5.2974000	11.3110000	27.5630000	10.0020000	13.8010000	3.1690000
2	***Adipoq***$^{-/-}$	56.2875000	72.9734000	67.2974000	71.2010000	50.3320000	60.0020000	14.1340000	13.3794000

	Group B			
	Adipoq$^{-/-}$			
	B:3	B:4	B:5	B:6
1	23.2940000	28.1120000	11.2120000	16.2790000
2	30.2740000	10.4340000	25.5560000	22.1102000

Table format: **Grouped**		Group A							
		Veh							
		A:1	A:2	A:3	A:4	A:5	A:6	B:1	B:2
1	**WT**	88.79954	84.78002	72.75676	77.21220	85.3340	81.65000	80.7359300	80.15436
2	***Adipoq*** $^{-/-}$	92.54530	93.55411	93.27897	94.21120	92.9980	91.22670	89.7024700	87.03297
3	***AdipoR1*** $^{-/-}$	94.96311	94.45683	92.74765	93.52134	95.1844	92.31150	86.6451800	86.70624
4	***AdipoR2*** $^{-/-}$	91.11496	89.70239	88.98521	90.28370	89.0306	87.93234	83.1556500	84.74576

	Group B			
	Ron			
	B:3	B:4	B:5	B:6
1	82.98109	79.4660000	86.0129000	75.8832000
2	89.05013	88.2134000	88.4311000	86.9910000
3	90.95411	87.4660000	91.1825000	86.4921000
4	84.42105	82.5653000	85.9031000	84.1131000

Table format: **Grouped**		Group A			Group B		
		**Veh**			**Ron**		
		A:1	A:2	A:3	B:1	B:2	B:3
1	**WT**	88.79954	84.78002	72.75676	80.73593	80.15436	82.98109
2	***Adipoq***$^{-/-}$	92.54530	93.55411	93.27897	89.70247	87.03297	89.05013
3	***AdipoR1***$^{-/-}$	94.96311	94.45683	92.74765	86.64518	86.70624	90.95411
4	***AdipoR2***$^{-/-}$	91.11496	89.70239	88.98521	83.15565	84.74576	84.42105

Table format: **Grouped**		Group A							
		Veh							
		A:1	A:2	A:3	A:4	A:5	A:6	B:1	B:2
1	**WT**	1.35000	2.09100	1.90000	5.287101	8.19370	15.2846293	1.578	2.320
2	***Adipoq*** $^{-/-}$	505.75496	511.28434	450.78000	472.172800	440.23740	428.6173000	265.142	289.317
3	***AdipoR1*** $^{-/-}$	412.38000	491.29300	371.28000	453.276390	392.13745	474.2738100	121.380	162.130
4	***AdipoR2*** $^{-/-}$	351.28000	421.28300	302.13000	410.389230	319.83740	394.2837640	130.293	121.382

	Group B			
	Ron			
	B:3	B:4	B:5	B:6
1	1.800	14.286492	7.29740	3.293749
2	242.167	321.286300	315.27450	256.242314
3	200.130	184.286210	146.27340	251.383640
4	110.293	151.837390	201.28374	193.283600

Table format: **Grouped**		Group A							
		Veh							
		A:1	A:2	A:3	A:4	A:5	A:6	B:1	B:2
1	**WT**	7.89000	5.21000	2.34000	1.34748	10.36480	15.29740	8.9860	6.987
2	***Adipoq*** $^{-/-}$	204.37390	210.38300	209.43874	215.23560	162.28360	179.28730	132.7650	125.643
3	***AdipoR1*** $^{-/-}$	179.39740	201.28300	163.27300	193.36820	171.28370	210.18373	109.3800	153.280
4	***AdipoR2*** $^{-/-}$	110.39000	89.32000	156.29830	131.23700	146.21700	95.27420	83.2973	74.293

	Group B			
	Ron			
	B:3	B:4	B:5	B:6
1	9.7540	12.46482	17.37927	2.26380
2	150.8643	193.29730	121.38690	204.28460
3	191.2830	215.28370	183.12370	174.29370
4	54.2740	69.39270	148.29340	91.13794

Table format: **Grouped**		Group A							
		Veh							
		A:1	A:2	A:3	A:4	A:5	A:6	B:1	B:2
1	**WT**	50.63500	58.08700	35.78600	47.28460	74.28640	45.27500	68.8530	54.7740
2	***Adipoq*** $^{-/-}$	739.37300	740.38000	746.48300	763.27400	667.29740	681.39740	328.2974	400.2937
3	***AdipoR1*** $^{-/-}$	621.28300	739.19000	681.30000	657.29740	721.37300	612.97170	271.2830	181.2380
4	***AdipoR2*** $^{-/-}$	982.18300	969.27390	821.38000	810.19340	881.36100	950.37290	421.3000	321.3400

	Group B			
	Ron			
	B:3	B:4	B:5	B:6
1	40.85460	69.29745	42.19745	28.19745
2	363.29748	430.18734	392.29740	482.19740
3	241.38300	194.18730	210.17300	159.19840
4	289.13400	317.38100	250.14980	350.09200

Table format: **Grouped**		Group A							
		Veh							
		A:1	A:2	A:3	A:4	A:5	A:6	B:1	B:2
1	**WT**	110.38300	91.12398	82.93700	86.39740	122.19730	95.29450	140.2930	130.7500
2	***Adipoq***$^{-/-}$	97.78000	121.86000	101.30000	118.37190	128.17390	110.38700	136.5770	135.2830
3	***AdipoR1***$^{-/-}$	92.29840	56.38490	78.38200	67.27100	83.13790	121.38300	131.2730	62.2840
4	***AdipoR2***$^{-/-}$	131.38400	69.29400	82.18300	72.13840	84.29470	113.29470	100.3830	82.2840

	Group B			
	Ron			
	B:3	B:4	B:5	B:6
1	140.83000	81.3984	91.3479	100.31398
2	137.38460	119.2740	102.1733	92.13830
3	84.29400	107.2973	75.4528	93.36450
4	93.82000	126.2133	77.2368	66.17340

Table format: **Grouped**		Group A							
		Veh							
		A:1	A:2	A:3	A:4	A:5	A:6	B:1	B:2
1	**WT**	156.28000	124.25000	144.36000	126.31000	134.11000	104.28000	107.22000	108.58000
2	***Adipoq*** $^{-/-}$	156.28400	167.71000	135.37000	195.82000	178.23000	129.18300	100.93000	93.82000
3	***AdipoR1*** $^{-/-}$	148.23000	102.85000	156.09000	133.84000	127.47000	99.18800	89.54000	107.22000
4	***AdipoR2*** $^{-/-}$	126.62000	132.33000	128.39000	140.76000	120.08100	110.28100	91.48000	104.69000

	Group B				Group C				
	Ron				Title				
	B:3	B:4	B:5	B:6	C:1	C:2	C:3	C:4	C:5
1	127.43000	147.67000	129.13000	108.12000					
2	113.19000	100.80000	118.39800	90.44800					
3	95.49000	121.31000	138.28000	124.77100					
4	115.21000	90.12110	99.17300	87.19300					

	C:6
1	
2	
3	
4	

Table format: **Grouped**		Group A							
		Veh							
		A:1	A:2	A:3	A:4	A:5	A:6	B:1	B:2
1	**WT**	44.29740	37.39750	48.39700	46.39750	29.13790	56.19830	30.39740	26.39740
2	***Adipoq*** $^{-/-}$	66.43000	70.29400	55.35000	65.34000	74.29700	69.33700	50.39850	43.93700
3	***AdipoR1*** $^{-/-}$	49.29400	48.39500	68.39500	47.35000	56.29872	54.29180	36.97500	41.39700
4	***AdipoR2*** $^{-/-}$	59.38500	42.93400	66.37500	50.37500	44.28871	58.82730	40.37500	48.49500

	Group B				Group C				
	Ron				Title				
	B:3	B:4	B:5	B:6	C:1	C:2	C:3	C:4	C:5
1	44.93740	39.37490	51.28300	22.19830					
2	36.37400	30.49500	50.18200	33.19380					
3	27.39750	50.37500	49.19000	55.54100					
4	45.47650	33.27400	49.11200	53.31300					

	C:6
1	
2	
3	
4	

Table format: **Grouped**		Group A							
		Veh							
		A:1	A:2	A:3	A:4	A:5	A:6	B:1	B:2
1	**WT**	51.38300	40.82000	58.07300	43.24000	37.23300	49.01200	32.28490	40.48390
2	***Adipoq*** $^{-/-}$	75.38400	69.79400	76.24200	69.12000	59.32200	73.36740	31.57000	46.47590
3	***AdipoR1*** $^{-/-}$	56.39750	49.38750	71.37930	63.29740	49.19180	51.27300	50.37400	60.38500
4	***AdipoR2*** $^{-/-}$	42.47900	59.37590	45.35900	48.38470	55.21100	39.47200	51.37480	39.49350

	Group B				Group C				
	Ron				Title				
	B:3	B:4	B:5	B:6	C:1	C:2	C:3	C:4	C:5
1	49.37490	45.39750	44.29300	39.97100					
2	35.38560	40.49370	45.81100	29.92800					
3	39.29740	40.37500	58.29820	45.21900					
4	47.74800	49.37500	46.28010	53.19830					

	C:6
1	
2	
3	
4	

	Group A	Group B	Group C	Group D
	NC	Sample	PC	IgG
1	779	610	603	204
2	739	581	579	216
3	756	621	590	198
4	762	600	587	187
5	770	619	595	203
6	745	590	573	220

	Group A	Group B	Group C	Group D	Group E
	WT	*Si rna WT*	*Adipor2$^{-/-}$*	*Adipor2$^{-/-}$*	*Adipor1$^{-/-}$*
1	5.54	0.293922427	1.28	0.120790330	0.18
2	5.93	0.313300000	1.49	0.218340000	0.28
3	6.03	0.419730000	2.19	0.161973400	0.22
4	5.78	0.278000000	2.78	0.319379000	0.12
5	6.09	0.438460000	1.83	0.286680000	0.33
6	5.24	0.355800000	1.59	0.188270000	0.21

Table format: **Grouped**		Group A							
		Ctr							
		A:1	A:2	A:3	A:4	A:5	A:6	B:1	B:2
1	**WT**	67.15953	68.10000	67.99000	68.23300	68.98800	67.54400	68.68867	67.42000
2	***Adipoq*** $^{-/-}$	58.47201	58.93790	57.19830	58.22100	57.92000	58.51398	55.01066	54.97200
3	***AdipoR2*** $^{-/-}$	73.59263	74.28600	73.13400	73.11200	75.93700	72.91100	68.56148	68.92740
4	***AdipoR1*** $^{-/-}$	54.71600	55.13874	54.92730	55.58270	54.91710	54.33890	50.28360	52.82630

	Group B AdipoRon				Group C Title				
	B:3	B:4	B:5	B:6	C:1	C:2	C:3	C:4	C:5
1	68.12830	68.927420	67.22460	67.14800					
2	55.39200	55.177000	54.22865	55.11420					
3	69.13974	68.944200	69.22480	68.82361					
4	51.13100	50.297287	51.99283	52.61930					

		Group D							
		Title							
	C:6	D:1	D:2	D:3	D:4	D:5	D:6	E:1	E:2
1								67.83468	67.12920
2								63.76689	63.12000
3								75.84481	74.24800
4									

	Group E				Group F				
	Adipor1 siRNA				*Adipor1* siRNA + AdipoRon				
	E:3	E:4	E:5	E:6	F:1	F:2	F:3	F:4	F:5
1	68.2930	67.93372	68.72648	68.18800	57.88091	56.27380	59.1863	56.29840	58.66438
2	64.0283	63.52810	64.99182	64.55230	60.43266	60.76300	61.9370	60.93740	61.34210
3	75.3862	75.52873	74.92200	75.18845	73.46939	73.92793	74.9273	74.15367	73.13500
4									

		Group G							
		Title							
	F:6	G:1	G:2	G:3	G:4	G:5	G:6	H:1	H:2
1	57.4832								
2	61.4234								
3	74.2643								
4									

	Group H				Group I				
	Title				Data Set-I				
	H:3	H:4	H:5	H:6	I:1	I:2	I:3	I:4	I:5
1					67.83468	67.1292	68.2930		
2					63.76689	63.1200	64.0283		
3					75.84481	74.2480	75.3862		
4									

		Group J							
		Data Set-J							
	I:6	J:1	J:2	J:3	J:4	J:5	J:6	K:1	K:2
1		67.93372	68.72648	68.18800				57.88091	56.27380
2		63.52810	64.99182	64.55230				60.43266	60.76300
3		75.52873	74.92200	75.18845				73.46939	73.92793
4									

	Group K				Group L				
	Data Set-K				Data Set-L				
	K:3	K:4	K:5	K:6	L:1	L:2	L:3	L:4	L:5
1	59.1863				56.29840	58.66438	57.4832		
2	61.9370				60.93740	61.34210	61.4234		
3	74.9273				74.15367	73.13500	74.2643		
4									

	L:6
1	
2	
3	
4	

	Group A	Group B	Group C	Group D	Group E
	**Veh**	**AdipoRon**		**Veh**	**AdipoRon**
1	4.2648290	5.91234000		0.1574490	0.91773400
2	3.2864800	2.04973400		0.4721560	1.07592100
3	5.2848600	6.23683100		0.1586040	1.63312200
4	5.1838000	6.92681900		0.3172980	1.72639000
5	4.6283000	8.27380000		0.5112300	2.32831000
6	3.9273913	7.26390000		0.3237400	2.31083000

	Group A	Group B	Group C	Group D	Group E
	**Veh**	**AdipoRon**		**Veh**	**AdipoRon**
1	74370.00000	79335.00000		71802.00000	81918.00000
2	74923.00000	79312.00000		71769.00000	81319.00000
3	73912.00000	79203.00000		71749.00000	81492.00000
4	75292.00000	80183.00000		73132.00000	82172.00000
5	73129.00000	78198.00000		72319.00000	80182.00000
6	74713.01830	78827.08930		71943.01000	81723.06627

	X	Group A	Group B	Group C
	Days	WT (n=9)	APKO (n=15)	Title
	X	Y	Y	Y
1	3	1		
2	2	1		
3	3	1	1	
4	4	1	1	
5	2			
6	3		1	
7	4		1	
8	5		1	
9	6		1	
10	7	0	0	
11	7	0	0	
12	7	0	0	
13	3	1	1	
14	3	1	1	
15	4	1	1	
16	4	1	1	
17	5		0	
18	5		0	

	Group A	Group B	Group C	Group D	Group E	Group F
	WT	***Adipoq*** $^{-/-}$	Title	Title	Title	Title
1	24.00	451.00				
2	6.00	75.00				
3	83.00	86.00				
4	7.00	567.00				
5	22.00	90.00				
6	4.00	241.00				
7	64.00	584.00				
8	7.00	74.00				
9	12.00	546.00				
10	7.00	37.00				

	Group A	Group B	Group C
	WT	***Adipoq***$^{-/-}$	Title
1	12.466670	11.67500	
2	11.266670	29.26667	
3	12.900000	18.80000	
4	13.933330	14.00000	
5	6.966670	23.35000	
6	6.880000	14.92000	
7	5.640000	15.86667	
8	6.075000	22.40000	
9	9.066667	11.36000	
10	5.600000	14.71000	

	Group A	Group B	Group C	Group D	Group E	Group F
	WT	APKO	Title	Title	Title	Title
1	426621.00	395959.00				
2	386721.00	425564.00				
3		424273.00				
4	371038.00	370522.00				
5		437253.00				
6		443220.00				
7		438198.00				
8		345241.00				
9						
10	387168.00					
11	335078.00					
12						
13	379339.00					

	Group A	Group B	Group C	Group D	Group E	Group F
	WT	***Adipoq***$^{-/-}$	Title	Title	Title	Title
1	45427.00	46185.00				
2	42067.00	36777.00				
3		38185.00				
4	35842.00	43151.00				
5		51150.00				
6		40457.00				
7		40165.00				
8		47913.00				
9						
10	38794.00					
11	35786.00					
12						
13	38374.00					

	Group A	Group B	Group C	Group D	Group E	Group F
	WT	***Adipoq***$^{-/-}$	Title	Title	Title	Title
1	6382.00	8782.00				
2	4687.00	6937.00				
3		7713.00				
4	4653.00	5869.00				
5		7477.00				
6		5948.00				
7		6757.00				
8		7153.00				
9						
10	3848.00					
11	3378.00					
12						
13	3543.00					

	Group A	Group B	Group C	Group D	Group E	Group F
	WT	***Adipoq*** $^{-/-}$	Title	Title	Title	Title
1	5981.00	8481.00				
2	10944.00	3456.00				
3		10878.00				
4	14522.00	7418.00				
5		7556.00				
6		2783.00				
7		9369.00				
8		6656.00				
9						
10	4027.00					
11	7539.00					
12						
13	11987.00					

	Group A	Group B	Group C	Group D	Group E	Group F
	WT	***Adipoq***$^{-/-}$	Title	Title	Title	Title
1	218336.00	163896.00				
2	225829.00	195709.00				
3		196269.00				
4	211017.00	131528.00				
5		224040.00				
6		233045.00				
7		238844.00				
8		129334.00				
9						
10	235778.00					
11	210422.00					
12						
13	237527.00					

Table format: **Grouped**		Group A WT							
		A:1	A:2	A:3	A:4	A:5	A:6	A:7	A:8
1	*Il-1α*	0.510000000	0.560000000	0.880000000		0.910000000	1.470000000		0.979810215
2	*Il-6*	0.410000000	0.400000000	0.970000000		1.140000000	2.080000000		0.941112070
3	*Il-10*	0.500000000	0.400000000	0.770000000		1.110000000	1.480000000		0.847484865
4	*Il-12β*								0.721798001
5	*Il-17α*	0.710000000	1.270000000	2.380000000		0.960000000	0.490000000		0.701531488
6	*Il-22*								0.800548228
7	*Tnf-α*	0.980000000	1.140000000	0.620000000		0.770000000	0.940000000		0.893397313

	A:9	A:10	A:11	A:12	A:13	A:14	B:1	B:2	B:3
1	0.876702324	1.164141790	1.017270868	0.662123741	1.929113529	0.769602457		3.750000000	6.620000000
2	0.854399761	1.243648179	0.798835261	0.656907998	2.500467538	0.762108846		5.380000000	9.060000000
3	0.813157371	1.451086981	0.701173359	0.731787790	1.850997612	1.052891393		2.260000000	3.650000000
4	0.968030739	1.431183027	0.879260697	0.962625616	0.823888326	1.434024515			
5	0.905295734	1.574571395	0.751320426	0.850177408	1.288781044	1.214747752		1.110000000	6.700000000
6	0.635001911	1.967149955	0.924936332	0.580215976	1.028882237	1.811059873			
7	0.825635240	1.355711053	0.967227023	0.822271842	1.156429186	1.087269276		2.830000000	1.900000000

	Group B								
	APKO								
	B:4	B:5	B:6	B:7	B:8	B:9	B:10	B:11	B:12
1	0.850000000		2.773661746	2.562386746	2.481039583		2.483872080	2.434673827	2.280337180
2	0.770000000		2.986299472	2.604514484	2.101469122		2.324811488	1.784302544	1.838949257
3	0.780000000		1.230899855	1.688429654	2.672527243		0.911984157	0.989281936	1.529427266
4			2.359684693	2.037234149	2.018583058		2.478759808	2.801305153	2.318652763
5	0.720000000		1.487224319	1.830172217	1.146452276		1.454387639	1.103070893	1.768464244
6			3.941865722	7.783353802	3.652108756		4.213272533	5.300639222	7.473720922
7	1.060000000		2.018296931	1.621783385	1.425094206		2.086342487	2.412457719	1.689662829

			Grou						
			Tr						
	B:13	B:14	C:1	C:2	C:3	C:4	C:5	C:6	C:7
1	2.428757502								
2	1.802232757								
3	2.225246441								
4	2.310381386								
5	1.028635759								
6	4.657080090								
7	1.530562431								

	up C								
	tle								
	C:8	C:9	C:10	C:11	C:12	C:13	C:14	D:1	D:2
1									
2									
3									
4									
5									
6									
7									

	Group D								
	Title								
	D:3	D:4	D:5	D:6	D:7	D:8	D:9	D:10	D:11
1									
2									
3									
4									
5									
6									
7									

	D:12	D:13	D:14	E:1	E:2	E:3	E:4	E:5	E:6
1									
2									
3									
4									
5									
6									
7									

	Group E								
	Title								
	E:7	E:8	E:9	E:10	E:11	E:12	E:13	E:14	F:1
1									
2									
3									
4									
5									
6									
7									

	Group F								
	Title								
	F:2	F:3	F:4	F:5	F:6	F:7	F:8	F:9	F:10
1									
2									
3									
4									
5									
6									
7									

	F:11	F:12	F:13	F:14
1				
2				
3				
4				
5				
6				
7				

Table format: **Grouped**		Group A							
		WT							
		A:1	A:2	A:3	A:4	A:5	A:6	B:1	B:2
1	*ADIPOR1*	1.50	0.99	1.02	0.82	0.83	0.97	1.35	1.13
2	*ADIPOR2*	1.83	0.85	1.09	0.88	0.86	0.78	1.50	1.10

	Group B			
	APKO			
	B:3	B:4	B:5	B:6
1	1.06	1.70	1.10	1.08
2	1.04	1.24	0.84	0.87

	Group A	Group B	Group C	Group D	Group E	Group F
	WT	***Adipoq***$^{-/-}$	Title	Title	Title	Title
1	0.069522241	0.072817133				
2	0.074464580	1.484678748				
3	0.453377265	0.342998353				
4	0.512685338	7.525864909				
5	0.046787480	14.57561779				
6	0.191762770	11.36803950[illegible]				

Table format: **Grouped**		Group A NI							
		A:1	A:2	A:3	A:4	A:5	A:6	A:7	A:8
1	*ADIPOQ*	1.48	0.62	0.80	0.60	1.58	1.43	1.49	0.92
2	*ADIPOR1*	1.12	0.69	1.17	1.01	1.02	1.07	1.17	1.01
3	*ADIPOR2*	1.12	0.86	1.06	0.84	0.97	1.20	1.23	1.08

	A:9	A:10	A:11	A:12	B:1	B:2	B:3	B:4	B:5
1	0.76	0.58	1.27	1.31	0.08	0.31	1.51	0.53	1.37
2	0.97	1.02	0.80	1.07	0.73	0.77	0.74	0.96	0.74
3	0.83	0.89	0.89	1.14	0.60	0.64	0.77	1.15	0.78

	Group B						
	Inf						
	B:6	B:7	B:8	B:9	B:10	B:11	B:12
1	0.68	0.10	0.28	1.28	0.48	1.13	0.54
2	1.13	0.88	0.73	0.74	0.94	0.66	0.85
3	0.98	0.66	0.61	0.65	0.65	0.62	0.68

Table format: **Grouped**		Group A							
		NI							
		A:1	A:2	A:3	A:4	A:5	A:6	B:1	B:2
1	*ADIPOQ*	0.6880240	1.45343760	1.0000000	1.3828340	1.9263890	1.31793000	2.9397775	3.9382135
2	*ADIPOR1*	1.0264000	0.97427900	1.0000000	0.9173000	1.0037802	0.83691734	2.7291763	2.4544511
3	*ADIPOR2*	1.0553332	0.94756800	1.0000000	0.9163740	1.1937930	0.71538400	0.3778497	0.3128936

	Group B			
	Inf			
	B:3	B:4	B:5	B:6
1	4.945333946	1.2746183	1.3793000	1.1737944
2	3.152332303	3.0183740	2.4626840	2.8163940
3	0.128669109	0.0183840	0.4158320	0.2169384

Table format: **Grouped**		Group A							
		NI							
		A:1	A:2	A:3	A:4	A:5	A:6	B:1	B:2
1	AMs	471	412		379	399	351	912	607
2	Eosinophils	151	144	146	216	276	254	285	230
3	$Cd11c^{+}$Siglec	403	542	322	507	393	332	756	597
4	$Cd11c^{-}$Siglec	382	272	467	350	392	265	375	338

	Group B Inf			
	B:3	B:4	B:5	B:6
1	754	552	519	605
2	254	299	300	300
3	664	497	392	556
4	331	407	394	380

	Group A	Group B	Group C	Group D
	WT	*Adipoq*$^{-/-}$	*Adipor1*$^{-/-}$	*Adipoq2*$^{-/-}$
1	1.113905	2.187429	0.150463	0.812376
2	1.116134	1.152110	0.000629	0.889471
3	0.946390	1.941122	0.181112	1.168096
4	1.575650	0.919222	0.217642	1.014193
5	0.799536	1.211793	0.338196	0.927390
6	0.674634	1.019987	0.156048	1.318734

	Group A	Group B	Group C	Group D	Group E
	**WT**	***Adipoq***$^{-/-}$	***Adipor1***$^{-/-}$	***Adipoq2***$^{-/-}$	Title
1	0.114522	8.030978	2.839784	1.500349	
2	1.472197	2.485000	1.461142	0.904806	
3	1.680205	4.466227	3.601035	2.495959	
4	2.254458	2.534974	4.042444	2.363002	
5	1.101854	2.145120	5.250389	1.917340	
6	1.421072	1.931338	3.124680	1.212970	

Table format: **Grouped**		Group A							
		Control							
		A:1	A:2	A:3	A:4	A:5	A:6	B:1	B:2
1	**WT**	2.95	3.21	3.00	4.72	3.61	4.01	13.30	15.60
2	***Adipoq***$^{-/-}$	31.50	29.80	30.12	28.65	32.13	28.96	0.12	4.12

	Group B			
	Infection			
	B:3	B:4	B:5	B:6
1	14.7	13.22	16.01	14.30
2	3.1	2.37	4.04	1.32

	Group A	Group B	Group C	Group D	Group E	Group F	Group G
	Control	Infection	ection+AdipoR		Control	Infection	ection+AdipoR
1	1.000000	0.057511728	0.417543960		0.109575715	0.075362989	0.108067154
2	1.000000	0.077481731	0.389582290		0.123279088	0.082469244	0.100830220
3	1.000000	0.058720172	0.307786103		0.133971683	0.089622203	0.110337875
4	1.000000	0.062874000	0.492837000		0.212837640	0.071910280	0.137900000
5	1.000000	0.079273000	0.392736000		0.172674000	0.083168390	0.183698000
6	1.000000	0.061934400	0.432792000		0.201378400	0.072028370	0.201736800

	Group A	Group B	Group C	Group D	Group E	Group F	Group G
	Control	Infection	ection+AdipoR		Control	Infection	ection+AdipoR
1	1.000000	0.023847800	0.222210670		0.035402621	0.024518253	0.027016788
2	1.000000	0.035158078	0.248273124		0.072795849	0.016288528	0.035402621
3	1.000000	0.026644840	0.190782401		0.056328154	0.012516717	0.026460791
4	1.000000	0.029237900	0.213930000		0.048297000	0.042937000	0.029237400
5	1.000000	0.031380000	0.301839000		0.028294700	0.031879300	0.031373900
6	1.000000	0.100378000	0.272983400		0.019739000	0.028284000	0.033864000

	Group A	Group B	Group C	Group D	Group E
	**Veh**	**AdipoRon**		**Veh**	**AdipoRon**
1	2.000000	1.00000000		1.0000000	1.00000000
2	2.000000	1.00000000		3.0000000	0.00000000
3	2.000000	2.00000000		2.0000000	1.00000000
4	3.000000	3.00000000		2.0000000	2.00000000
5	2.000000	2.00000000		3.0000000	0.00000000
6	1.000000	2.00000000		3.0000000	0.00000000
7	1.000000	3.00000000		3.0000000	0.00000000
8	1.000000	3.00000000		3.0000000	0.00000000
9	1.000000	3.00000000		3.0000000	1.00000000
10	2.000000	2.00000000		3.0000000	1.00000000

	Group A	Group B	Group C
	Day 10	Day 15	Day 25
1	98.1	99.2	98.3
2	97.2	99.6	97.5
3	98.8	98.1	99.1
4	99.4	99.5	98.4
5	97.1	99.8	97.6
6	98.6	98.6	98.7

	Group A	Group B	Group C
	Day 10	Day 15	Day 25
1	97.1	99.1	96.9
2	98.9	98.3	99.6
3	97.4	99.5	98.1
4	98.5	99.2	98.6
5	97.5	98.1	96.4
6	98.9	98.4	97.4

	Group A	Group B
	AMs	BMDMs
1	0.062937	0.02000
2	0.091900	0.01800
3	0.071200	0.03800
4	0.082620	0.04869
5	0.056110	0.03172

	Group A	Group B
	AMs	BMDMs
1	5.82300	0.001
2	4.32000	0.020
3	8.38400	0.006
4	3.92680	0.040
5	2.17293	0.003
6	2.52872	0.005

	Group A	Group B	Group C
	Data Set-A	Data Set-B	Title
1	0.8	6.1	
2	1.1	5.7	
3	0.6	5.1	
4	1.5	6.3	
5	0.7	5.5	
6	0.6	5.9	

	Group A	Group B	Group C
	Data Set-A	Data Set-B	Title
1	2.7340	7.92100	
2	3.1220	6.83400	
3	1.2430	7.19928	
4	3.8261	5.82400	
5	3.4140	4.26110	
6	3.4010	4.71920	

Table format: **Grouped**		Group A							
		Veh							
		A:1	A:2	A:3	A:4	A:5	A:6	B:1	B:2
1	WT	2.5840	4.28460	6.29730	4.92900	5.27938	3.29184	3.2974	6.2846
2	Adipoq$^{-/-}$	1.3974	0.62974	2.29400	3.92682	1.83680	3.13830	4.2749	5.1347

	Group B				Group C				
	Ron				Title				
	B:3	B:4	B:5	B:6	C:1	C:2	C:3	C:4	C:5
1	6.92100	3.1500	4.29109	3.92726					
2	4.72984	3.9279	5.72920	5.31930					

		Group D					
		Title					
	C:6	D:1	D:2	D:3	D:4	D:5	D:6
1							
2							
