# Supplementary material for: Adiponectin pathway activation dampens inflammation and enhances alveolar macrophage fungal killing via LC3-associated phagocytosis
Source: PLoS Pathog. 2025 Mar 17;21(3):e1012363. doi: 10.1371/journal.ppat.1012363 (PMC11949351; doi:10.1371/journal.ppat.1012363)
Supplement: S1 Text — Methods for supporting data figures. (DOCX) [file ppat.1012363.s010.docx]

**Supplemental Methods (See also Key Resources (S1 Table))**

**Mice**

An additional strain of *Adipoq*-/- mice was obtained from Dr. Philipp Scherer (University of Texas-Southwestern) and used for survival, fungal burden, histology, flow cytometric analysis of BALF cells, qRT-PCR of selected inflammatory and *Adipor* genes, and ELISA for TNF as described in the *Materials and Methods* section.

**Quantification of AM AdipoR1 expression using flow cytometry**

After ex-vivo AM extraction and culture as mentioned in the methods, AMs were fixed using IC fixation buffer. Fc Block was used to eliminate non-specific Fc-mediated interactions. The cells were then stained at a 1:100 dilution with primary antibody with Adiponectin Receptor 1 Recombinant Rabbit Monoclonal Antibody (SC69-04) followed by staining with Alexa Fluor 488-conjugated goat anti rabbit IgG as the secondary antibody. All the unstained and only primary antibody staining controls were included.

**Quantification of AM *Adipor1* and *Adipor2* receptor gene expression**

RNA extraction from AMs was done using Qiagen RNeasy Mini kit, following the manufacturer’s protocol. Quantitative RT-PCR was performed with 20ng of cDNA using AdipoR1 and AdipoR2 forward and reverse primers ordered from IDT Integrated Technologies. Gene Expression Master Mix (ThermoFisher Scientific) was used, with β-actin used for signal normalization.

**RNA sequencing and analysis**

**Sample Preparation**

AMs were isolated from APN-/- mice, infected/uninfected for 10 hours (1:9 cells/conidia) and AdipoRon/vehicle treated 24 hours before infection, with cells lysed and RNA isolated as described in *Materials and Methods*.

**Library preparation and sequencing**

Total RNA samples were first evaluated for their quantity and quality using Agilent TapeStation. All the samples were good quality with RIN (RNA Integrity Number) of 9.6-10. One hundred nanograms of total RNA was used for library preparation with the Illumina Stranded mRNA Prep, Ligation kit (lllumina), following the manufacturer’s instruction. Each resulting uniquely dual-indexed library was quantified and quality accessed by Qubit and Agilent TapeStation, and multiple libraries were pooled in equal molarity. The pooled libraries were sequenced with 2×150bp paired-end configuration on an Illumina NovaSeq X PLUS sequencer.

**RNA-seq data analysis**

The sequencing reads were first quality checked using FastQC (v.0.11.5, Babraham Bioinformatics, Cambridge, UK) for quality control. The sequence data were then mapped to the mouse reference genome mm10 using the RNA-seq aligner STAR (v.2.7.10a)[1] with the following parameter: “--outSAMmapqUnique 60”. To evaluate quality of the RNA-seq data, the number of reads that fell into different annotated regions (exonic, intronic, splicing junction, intergenic, promoter, UTR, etc.) of the reference genome was assessed using bamutils (from ngsutils v.0.4.17) [2]. Uniquely mapped reads were used to quantify the gene level expression employing featureCounts (subread v.2.0.3) [3] with the following parameters: “-s 2 -Q 10”. The data was normalized using TMM (trimmed mean of M values) method. Differential expression analysis was performed using edgeR (v.4.0.1)[3,4]. False discovery rate (FDR) was computed from p-values using the Benjamini-Hochberg procedure. Gene Ontology (GO) pathway enrichment analyses were performed with the R package [clusterProfiler](https://yulab-smu.top/biomedical-knowledge-mining-book/index.html) [5,6].

**Histopatholocial analysis**

Lung tissues were harvested 3 days post infection with conidia from vehicle or AdipoRon-treated mice and stained with hematoxylin-eosin (H&E). To examine the extent of lung injury, we considered its five pathological features, including (i) presence of exudates, (ii) hyperemia/congestion, (iii) intra-alveolar hemorrhage/debris, (iv) cellular infiltration, and (v) cellular hyperplasia. The severity of each of pathological features was evaluated by a score indicating 0 as absent/none, 1 as mild, 2 as to show moderate, and 3 for severe injury.

**ELISA for LC3-II**

After culturing for 10 days, AMs were grouped into vehicle (DMSO) + infected and AdipoRon + infected groups. The timeline was followed as per in Fig. 7E, followed by LC3-II (Cat. # CBA-5116, Cell Biolabs Inc (California, USA)) detection at an absorbance of 450nm, as per the manufacturer instructions for commercial kits.

**References**

1. Dobin A, Davis CA, Schlesinger F, Drenkow J, Zaleski C, Jha S, et al. STAR: ultrafast universal RNA-seq aligner. Bioinformatics. 2013;29: 15–21. doi:10.1093/BIOINFORMATICS/BTS635

2. Liao Y, Smyth GK, Shi W. featureCounts: an efficient general purpose program for assigning sequence reads to genomic features. Bioinformatics. 2014;30: 923–930. doi:10.1093/BIOINFORMATICS/BTT656

3. Robinson MD, McCarthy DJ, Smyth GK. edgeR: a Bioconductor package for differential expression analysis of digital gene expression data. Bioinformatics. 2010;26: 139–140. doi:10.1093/BIOINFORMATICS/BTP616

4. McCarthy DJ, Chen Y, Smyth GK. Differential expression analysis of multifactor RNA-Seq experiments with respect to biological variation. Nucleic Acids Res. 2012;40: 4288–4297. doi:10.1093/NAR/GKS042

5. Yu G, Wang LG, Han Y, He QY. clusterProfiler: an R package for comparing biological themes among gene clusters. OMICS. 2012;16: 284–287. doi:10.1089/OMI.2011.0118

6. Wu T, Hu E, Xu S, Chen M, Guo P, Dai Z, et al. clusterProfiler 4.0: A universal enrichment tool for interpreting omics data. Innovation (Cambridge (Mass)). 2021;2. doi:10.1016/J.XINN.2021.100141
